# Isocyanonaphthol Derivatives: Excited-State Proton Transfer and Solvatochromic Properties

**DOI:** 10.3390/ijms23137250

**Published:** 2022-06-29

**Authors:** Anita Adamoczky, Tibor Nagy, Péter Pál Fehér, Veronika Pardi-Tóth, Ákos Kuki, Lajos Nagy, Miklos Zsuga, Sándor Kéki

**Affiliations:** 1 Department of Applied Chemistry, Faculty of Sciences and Technology, University of Debrecen, Egyetem tér 1, H-4032 Debrecen, Hungary; adamoczky.anita@science.unideb.hu (A.A.); nagy.tibor@science.unideb.hu (T.N.); pardi-toth.veronika.csilla@science.unideb.hu (V.P.-T.); kuki.akos@science.unideb.hu (Á.K.); nagy.lajos@science.unideb.hu (L.N.); zsuga.miklos@science.unideb.hu (M.Z.); 2 Doctoral School of Chemistry, University of Debrecen, Egyetem tér 1., H-4032 Debrecen, Hungary; 3 Research Centre for Natural Sciences, Magyar Tudósok Körútja 2., H-1117 Budapest, Hungary; feher.peter@ttk.mta.hu

**Keywords:** 5-isocyanonaphthalene-1-ol, 5-isocyano-1-(octyloxy)naphthalene, fluorescence, dual emission, excited-state proton transfer, photoacid

## Abstract

Fluorescent probes that exhibit solvatochromic or excited-state proton-transfer (ESPT) properties are essential tools for the study of complex biological or chemical systems. Herein, the synthesis and characterization of a novel fluorophore that reveals both features, 5-isocyanonaphthalene-1-ol (ICOL), are reported. Various solvatochromic methods, such as Lippert–Mataga and Bilot–Kawski, together with time-dependent density functional theory (TD-DFT) and time-resolved emission spectroscopy (TRES), were applied to gain insights into its excited-state behavior. To make comparisons, the octyloxy derivative of ICOL, 5-isocyano-1-(octyloxy)naphthalene (ICON), was also prepared. We found that internal charge transfer (ICT) takes place between the isocyano and –OH groups of ICOL, and we determined the values of the dipole moments for the ground and excited states of both ICOL and ICON. Furthermore, in the emission spectra of ICOL, a second band at higher wavelengths (green emission) in solvents of higher polarities (dual emission), in addition to the band present at lower wavelengths (blue emission), were observed. The extent of this dual emission increases in the order of 2-propanol < methanol < *N*,*N*-dimethylformamide (DMF) < dimethyl sulfoxide (DMSO). The presence of the dual fluorescence of ICOL in these solvents can be ascribed to ESPT. For ICOL, we also determined ground- and excited-state pK_a_ values of 8.4 ± 0.3 and 0.9 ± 0.7, respectively, which indicates a considerable increase in acidity upon excitation. The TRES experiments showed that the excited-state lifetimes of the ICOL and ICON spanned from 10.1 ns to 5.0 ns and from 5.7 ns to 3.8 ns, respectively. In addition, we demonstrated that ICOL can be used as an effective indicator of not only the critical micelle concentration (cmc) of ionic (sodium lauryl sulfate (SLS)) and nonionic surfactants (Tween 80), but also other micellar parameters, such as partition coefficients, as well as to map the microenvironments in the cavities of biomacromolecules (e.g., BSA). It is also pointed out that fluorescence quenching by pyridine can effectively be utilized for the determination of the fractions of ICOL molecules that reside at the water–micelle interface and in the interior spaces of micelles.

## 1. Introduction

Molecules that respond to stimuli induced by their environments in a controlled manner can be used as probes to study the properties of various complex supramolecular chemical and biological systems, such as micelles, liposomes or biomacromolecules [1]. Due to the high sensitivity and accuracy required in these studies, the introduction of novel probes, in addition to the continuous improvements in the existing ones, is the focus of the current materials research [2,3]. Most of these compounds are fluorescent [4]; thus, the changes observed in their emission spectra may reveal information about the polarity [5], acidity/basicity [6] or even the fluidity of their microenvironments [7]. Fluorescent dyes that change their emission properties with the polarity of their microenvironment are termed solvatochromic dyes, and they play an important role in the localization of the hydrophobic and/or hydrophilic cavities of large biomolecules, such as bovine serum albumin (BSA) [8,9]. Most of the dyes employed for such purposes exhibit a positive solvatochromic effect, which refers to an increase in the dipole moment of the excited state with respect to that of the ground state, which results in a redshift of the emission spectrum with increasing solvent polarity [10]. Fluorescent dyes that exhibit positive solvatochromism are often called “push-pull” dyes, as they contain electron-donor (e.g., amine) and electron-acceptor (e.g., formyl) groups that are connected through a conjugated linker (i.e., an aryl group, such as naphthalene) [11]. The acidity or basicity of a complex chemical system also plays a decisive role in the proton-transfer reactions that occur in various chemical and biochemical reactions, serving also as the fundamental process for, for example, the bioenergetics of proteins and lipid bilayers [12,13]. Compounds in which the acidity (value of K_a_) in the excited state can increase by a factor of 10^5^−10^8^ or more with respect to that of the ground state are called photoacids [14]. These compounds, such as the aromatic hydroxy compounds, undergo excited-state proton transfer (ESPT) upon irradiation by light, and they seem to be ideal candidates for probing acidity/basicity through ESPT processes, or for inducing a pH drop in their microenvironments [15,16,17,18,19,20,21,22,23]. Furthermore, photoacids can potentially be applied in proton-catalyzed photochemical reactions, or for controlling the enzyme activity by a light-induced pH jump to initiate biochemical reactions [15,24,25].

Recently, we developed a new family of solvatochromic dyes called ICAN (isocyano-aminonaphthalene), based on internal charge transfer (ICT), by introducing electron-donor amino (-NH_2_) and electron-withdrawing isocyano (-N^+^≡C^−^) groups into the naphthalene ring [26,27,28]. Based on the same concept, additional solvatochromic dyes, including 3-amino-6-isocyanoacridine, were also developed, in which the acidity was fine-tuned to near the physiological pH (pK_a_ = 7.05–7.58) [29]. Our studies also demonstrated that the most pronounced solvatochromic response can be achieved by placing the donor and acceptor groups into the 1,5-positions of the naphthalene core (1,5-ICAN) [30]. Thus, we envisioned that replacing the amino group in 1,5-ICAN with an –OH moiety (5-isocyanonaphthalene-1-ol (ICOL)) would also exhibit a significant solvatochromic effect due to the similarity of the electron-donating character of the -OH and amino groups. This change is also expected to make ICOL a photoacid that is more potent than the generally applied 1-naphthol, which is due to the presence of the electron-withdrawing isocyano group. In this work, we show that this combination of solvatochromism and photoacidity can provide a unique way to obtain information not only on the polarity, but also on the acidity/basicity of the microenvironments around ICOL molecules. In addition to ICOL, we also report the synthesis and detailed characterization of its octyloxy derivative, 5-isocyano-1-(octyloxy)naphthalene (ICON). Although the emission properties of some cyanonaphtholes have already been studied [31,32], those of the isocyanonaphthole derivatives, to the best of our knowledge, have not yet been explored.

## 2. Result and Discussion

### 2.1. UV–Vis and Fluorescence Properties of isocyanonaphthalene-1-ol (ICOL) and 5-isocyano-1-(octyloxy)naphthalene (ICON)

**Figure 1:**
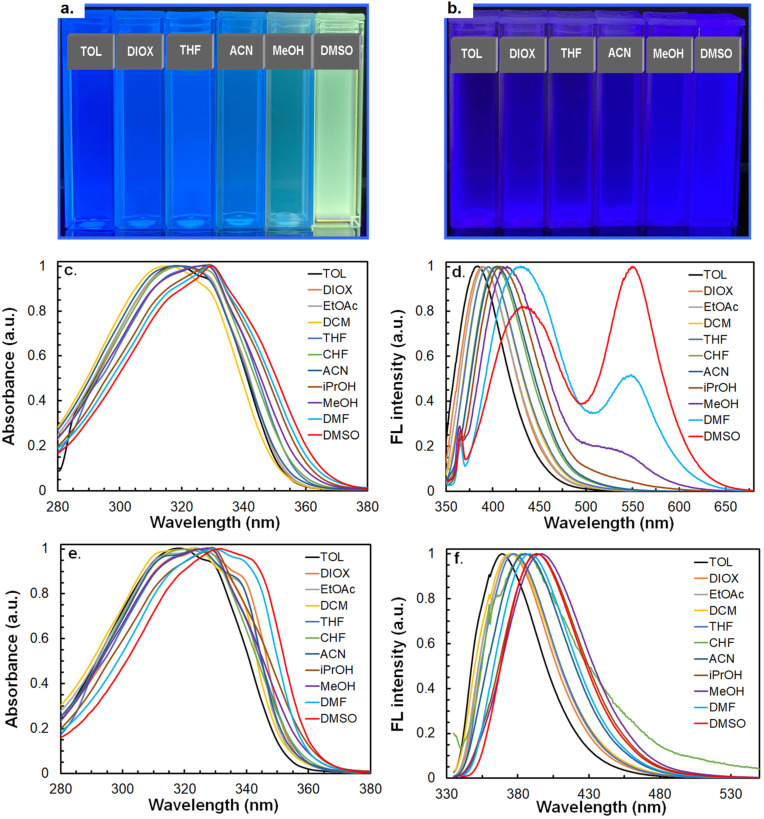
Visualization of the fluorescence of (**a**) 5-isocyanonaphthalene-1-ol (ICOL) and (**b**) 5-isocyano-1-(octyloxy)naphthalene (ICON). UV–Vis and fluorescence spectra of (**c**,**d**) ICOL and (**e**,**f**) ICON dissolved in various solvents: TOL: toluene; DIOX: 1,4-dioxane; EtOAc: ethyl acetate; DCM: dichloromethane; THF: tetrahydrofuran; CHF: chloroform; ACN: acetonitrile; iPrOH: 2-propanol; MeOH: methanol; DMF: *N*,*N*-dimethylformamide; DMSO: dimethyl sulfoxide.

The electronic absorption (UV–Vis) and emission properties of isocyanonaphthalene-1-ol (ICOL) and isocyano-1-(octyloxy)naphthalene (ICON) were investigated in various solvents with different polarities and proton donor/acceptor properties. The corresponding UV–Vis and emission spectra are shown in Figure 1, whereas the measured optical properties for the ICOL and ICON solutions are compiled in Table 1 and Table 2, respectively.

**Figure 2:**
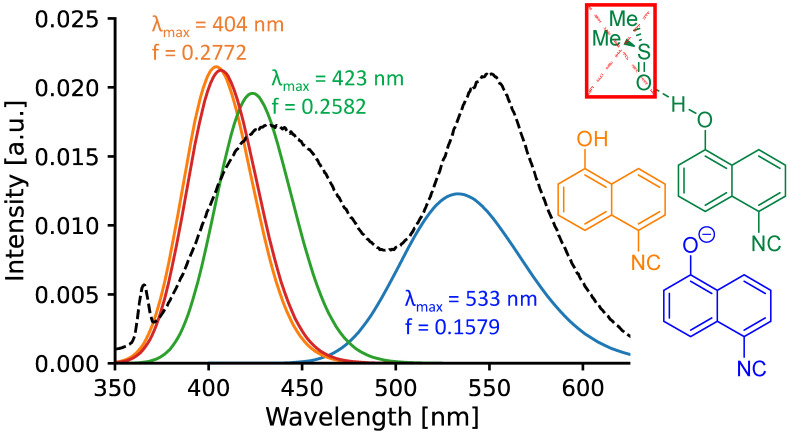
The calculated emission spectra of 5-isocyanonaphthalene-1-ol (ICOL) in dimethyl sulfoxide (DMSO), showing only the first emission peaks for all indicated species. The orange and blue lines correspond to the neutral and deprotonated forms of ICOL, respectively, using only implicit solvation. The green line corresponds to the ICOL–DMSO adduct. The red line corresponds to the case where the DMSO was removed from the adduct while keeping the geometry of the ICOL molecule unchanged. The dashed black line corresponds to the experimental spectrum, which is rescaled to match the intensity range of the calculated spectra.

As seen in Figure 1, and from the data in Table 1 and Table 2, both ICOL and ICON exhibit a positive solvatochromic effect (i.e., the absorption electronic and emission spectra redshift upon increasing the solvent polarity). The excitation-maxima and molar-absorption coefficients for ICON are higher than those obtained for ICOL due to the positive inductive effect of the long alkyl chain (octyl). On the contrary, the emission maxima for ICOL are shifted from 385 nm to 435 nm, while, for ICON, they span only from 368 nm to 395 nm, as they go from toluene to dimethyl sulfoxide (DMSO). This observation may indicate that the naphtholic hydroxyl group can interact with the surrounding solvent molecules more effectively in the excited state, thereby reducing the energy gap between the ground and excited states. Furthermore, it can also be concluded from the data in Table 1 and Table 2 that the quantum yield for ICOL decreases considerably with the solvent polarity; for example, the value of the Q_F_ in tetrahydrofuran (THF) (0.69) reduces to 0.07 in *N*,*N*-dimethylformamide (DMF). The smaller Q_F_ values in polar solvents can be ascribed to the narrower energy gap between the ground and excited states that is associated with an increased nonradiative decay rate in these solvents. On the contrary, no similar solvent polarity effect could be established for ICON. One of the most intriguing findings, however, is the dual emission of ICOL in polar solvents. The 1-naphthol also exhibits dual emission but, due to the presence of the electron-withdrawing isocyano group in ICOL, its second emission band appears at a longer wavelength (550 nm) compared with that of 1-naphthol (460 nm) [33]. Furthermore, as seen in Figure 1d, the intensity of the band at around 550 nm increases relative to that of the band at a shorter wavelength in the order of 2-propanol < methanol < DMF < DMSO, and it is also visualized by the color change shown in Figure 1a. As no such dual emission was found in the case of the ICON solutions, the band at the longer wavelength can, similar to the case of the 1-naphthol, be attributed to the emission from the anion (i.e., isocyanonaphtholate ion) generated via the excited-state proton transfer (ESPT). This is supported by the time-dependent density functional theory (TD-DFT) results shown in Figure 2. The calculated spectra indicate that a simple ICOL–DMSO interaction cannot explain the large peak separation, and that the observed emissions at 550 nm can be attributed to the deprotonated ICOL.

**Figure 3:**
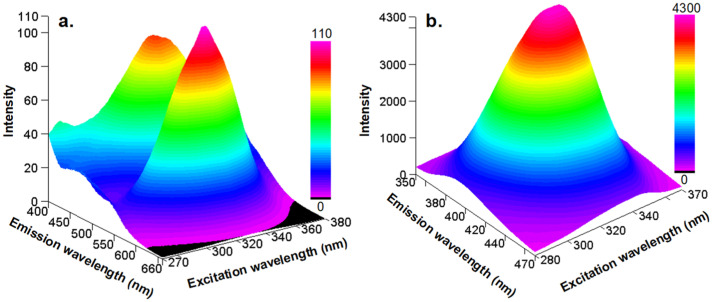
3D fluorescence mapping of (**a**) 5-isocyanonaphthalene-1-ol (ICOL) and (**b**) 5-isocyano-1-(octyloxy)naphthalene (ICON), dissolved in dimethyl sulfoxide (DMSO).

**Figure 4:**
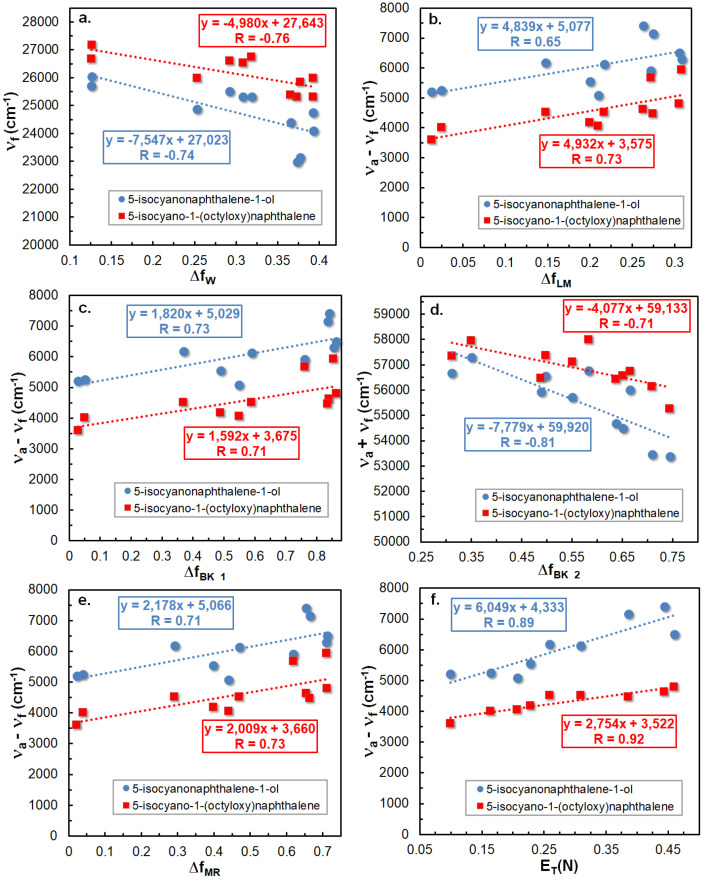
The (**a**) Weller, (**b**) Lippert–Mataga, (**c**,**d**) Bilot–Kawski, (**e**) McRea and (**f**) Reichardt plots for 5-isocyanonaphthalene-1-ol (ICOL) and 5-isocyano-1-(octyloxy)naphthalene (ICON) dissolved in various solvents. Δf_w_, Δf_LM_, Δf_BK_1_, Δf_BK_2_ and Δf_MR_ are the Weller, Lippert–Mataga, Bilot–Kawski 1, Bilot–Kawski 2 and McRea solvent polarity functions. E_T_(N) stands for the normalized Reichardt’s parameter, and R is the correlation coefficient.

In order to shed more light on the fluorescence properties of ICOL and ICON, 3D fluorescence spectra were recorded. As it turns out from Figure 3, no new band appears upon changing the excitation wavelength for ICON (Figure 3b), which indicates the presence of a single emitting species. Furthermore, it can also be observed for the DMSO solution of ICOL (Figure 3a) that the intensity of the emission band at a longer wavelength band varies simultaneously with that of the shorter wavelength upon changing the excitation wavelength. This latter finding suggests that excitation occurs on a single species, but emissions come from two different species. In the following, we focus our studies on the solvatochromic properties of ICOL and ICON. To evaluate the effects of different solvents on the emission behaviors of ICOL and ICON, and to estimate the ground (μ_G_)- and excited (μ_E_)-state dipole moments, the various solvatochromic shift methods of Weller [23], Lippert–Mataga [34,35], Bilot–Kawski [36,37], McRea [38] and Reichardt [39] were employed. According to these methods, the spectral characteristics (e.g., the Stokes shifts, or the wavenumbers at maximum emission intensities) can be plotted as a function of the corresponding solvent polarity function, and, from the linear fits to the experimental data, the specific dipole moments can be extracted. These plots, together with the fitted lines, are presented in Figure 4.

All the data and equations used to obtain and analyze the evaluations of the plots shown in Figure 4 are summarized in the Supplementary Material (Equations (S1)–(S17), Table S1). As Figure 4 shows, the linear fits with moderate correlation coefficients were obtained, and the estimated dipole moments from the slopes of these plots are compiled in Table 3.

**Figure 5:**
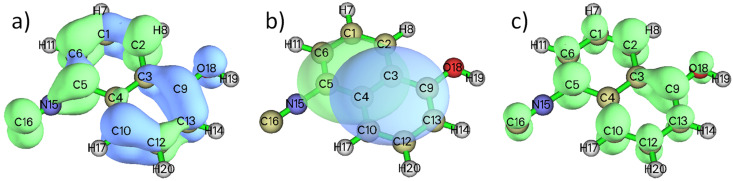
Isosurfaces calculated for (**a**) the hole (blue) and electron (green) distributions, (**b**) the C_hole_ and C_ele_ functions and (**c**) the hole and electron overlap (S_r_) function of 5-isocyanonaphthalene-1-ol (ICOL) in toluene. An isovalue of 0.002 electrons/bohr^3^ was used for all three surfaces.

According to the data presented in Table 3, all the applied models agree that the ICOL and ICON molecules exhibit almost identical dipole changes (Δµ) upon excitation. The Bilot–Kawski, McRea and Riechardt methods yielded similar Δµ values (3.2 D–3.7 D, except for ICON), while the Lippert–Mataga approach predicted higher values of Δμ for both ICOL and ICON (i.e., 5.5 D and 5.6 D, respectively). The µ_G_, µ_E_ and Δµ values were also calculated with the TD-DFT, and the results agree with the empirical Bilot–Kawski model for the ICOL molecule. In the case of ICON (in these calculations, the octyl group was replaced by a methyl group), however, the DFT calculations predicted significantly higher dipole moments. This can be explained by the presence of an internal-charge-transfer (ICT) interaction. Figure 5 and Figure S7 indicate that, during vertical excitation, a part of the π-electron density that is present on the phenol side of the ICOL molecule is transferred to the isocyano side. To quantify the extent of this ICT, the Δr and Λ parameters were also calculated. The Δr value of 1.1784 Å and Λ index of 0.7933 both indicate a weak charge transfer, as the first measures the length of the excitation, and the second is related to the overlap between the hole and electron distributions. The two values were also calculated in DMSO, where Δr = 1.5525 Å and Λ = 0.7422 were obtained, which indicate an only slightly stronger interaction. In the ICON derivative, a similar kind of charge transfer can be expected, where the addition of an electron-donating alkyl substituent to the −OH group increases the electron density of the phenol side. From this, it follows that ICON should have a higher ground-state dipole and stronger ICT interaction. Although this explains the larger calculated dipoles in Table 3, the deviation from the empirical models is likely due to an additional solute–solvent interaction, or to entropic effects that were not accounted for in the DFT calculations.

Multilinear regression analysis (MLR), as proposed by Kamlet and Taft (KT) [40,41], was also performed to find out the effects of the specific and nonspecific solute–solvent interactions on the fluorescence properties of ICOL and ICON. The KT parameters, such as the fluorescence wavenumbers of the gas-phase π-π* electronic transition (ν_o_), hydrogen-bond-donor capacity (a_α_), hydrogen-bond-acceptor capacity (b_β_) and polarity/polarizability (c_π*_), were estimated by the fitting of Equation (1) to the experimental data:

(1) $$Y=Y_{o}+\alpha a_{\alpha }+\beta b_{\beta }+\pi^{*}c_{\pi *}$$

where Y represents the observed emission maximum (ν_F_) or the Stokes shift (Δν); Y_o_ is the emission maximum (ν_o_) or the Stokes shift (Δν_o_) in the gas phase; α, β and π* stand for the acidity, basicity and polarity/polarizability of the solvents, respectively. The solvent parameters used for the estimation are compiled in the Supplementary Materials (Table S2).

On the one hand, as it turns out from the data in Table 4, all the solvent parameters in Equation (1) for the ν_F_-s are negative, which confirms that all of them contributed to the observed redshifts in the emission spectra. On the other hand, as judged from the largest absolute c_π*_ values, it can be concluded that the nonspecific solute–solvent interactions had the largest effect on the emission spectra of both ICOL and ICON. In addition, the higher absolute values of the b_β_ (1730 cm^−1^ and 1305 cm^−1^) with respect to those of the a_α_ (615 cm^−1^ and 1050 cm^−1^) confirm that the basicity of the solvent primarily determines the bathochromic shifts of ICOL and ICON.

**Figure 6:**
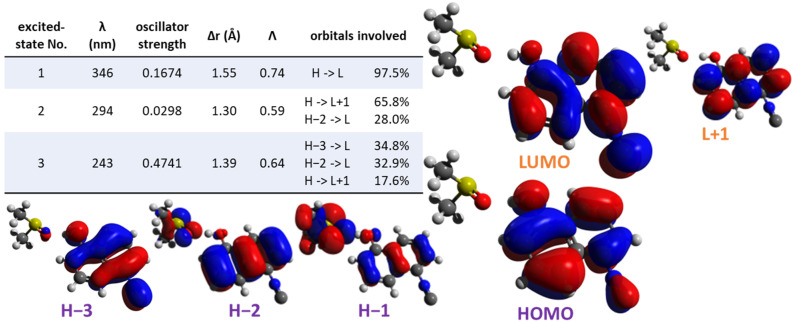
The frontier orbitals and calculated parameters of the first three excitations of the 5-isocyanonaphthalene-1-ol– dimethyl sulfoxide (ICOL–DMSO) adduct in DMSO.

The interaction between ICOL and DMSO was studied by the TD-DFT through the calculation of the emission spectra using both implicit and explicit solvation. When only implicit solvation was applied, the emission maximum was found at 404 nm. By adding an explicit DMSO molecule hydrogen bonded to the –OH moiety of ICOL, the emission maximum shifted to 423 nm, which is close to the observed one at 435 nm (Figure 2 and Figure S8). The origin of this redshift can be separated into two parts: the elongation of the naphtholic O–H bond of ICOL, and the presence of the H-bond acceptor DMSO. The former has an almost negligible effect (3 nm shift) on the emission wavelength; therefore, the presence of the DMSO molecule accounts for most of the overall 19 nm redshift between the single ICOL molecule and its DMSO adduct. To evaluate the nature of this interaction, a molecular orbital analysis was carried out (Figure S9). The TD-DFT calculations indicate that the first excitation contains, almost exclusively, a local HOMO-to-LUMO (Figure 6) excitation. This, together with the wavelength shifts discussed above, indicate a specific solute–solvent interaction between the neutral form of ICOL and the DMSO, which does not involve charge transfer, but only the polarization and hydrogen-bond interaction between the two molecules. We also calculated the emission spectra of ICOL in toluene using implicit solvation (Figure S10, Table S7). The results indicate that no ESPT takes place, as the emission comes from only the neutral ICOL at 387 nm, which is in good agreement with the experimental observations. A comparison of the calculated (387 nm) and measured (384 nm) wavelengths of the peak maxima also indicates that the effect of the solvent–solute interaction in toluene is negligible (Figures S12 and S13, Table S5).

### 2.2. Acidity and Photoacidity of ICOL Derivative

As mentioned earlier, the dual emission of ICOL in polar solvents is due to the fluorescence of the neutral form of ICOL at lower wavelengths, while emission at longer wavelengths is attributable to the anionic form of ICOL (i.e., to the isocyanonaphtholate ion). However, ICOL weakly fluoresces in water, and only a low-intensity band at 540 nm with a quantum yield of about 0.007 is present, which indicates the absence of the neutral form of ICOL. To determine the ground-state and excited-state pK_a_ values of ICOL, the electronic absorption and emission spectra of ICOL at different pH values were recorded and evaluated according to the procedure presented in the Supplementary Material. The ground-state and excited-state values of the pK_a_ for the ICOL were determined to be 8.4 ± 0.3 and 0.9 ± 07 (using Equation (S25)), respectively (Figures S14 and S15). The excited-state pK_a_^*^ was also estimated using the Förster cycle [20] (Equation (S26)), which yielded a value of 0.07, which is in fairly good agreement with the value determined experimentally. The reported values of the pK_a_ for 1-naphthol are 9.4 and 0.5 [42]. Hence, our experiments also pointed out that, by introducing the electron-withdrawing isocyano group into the naphthol, the acidities of both the ground and excited states can be increased simultaneously. Similar effects were also reported for the cyanonaphthols [31,32]. The details of the determination of the pK_a_ values are summarized in the Supplementary Materials. It is to be noted, however, that the determined value of the pK_a_* can be considered only as an apparent value because the proton-induced quenching of the anionic form can take place at higher (H^+^) [33].

### 2.3. Preferential Solvation of ICOL and ICON Derivatives

**Figure 7:**
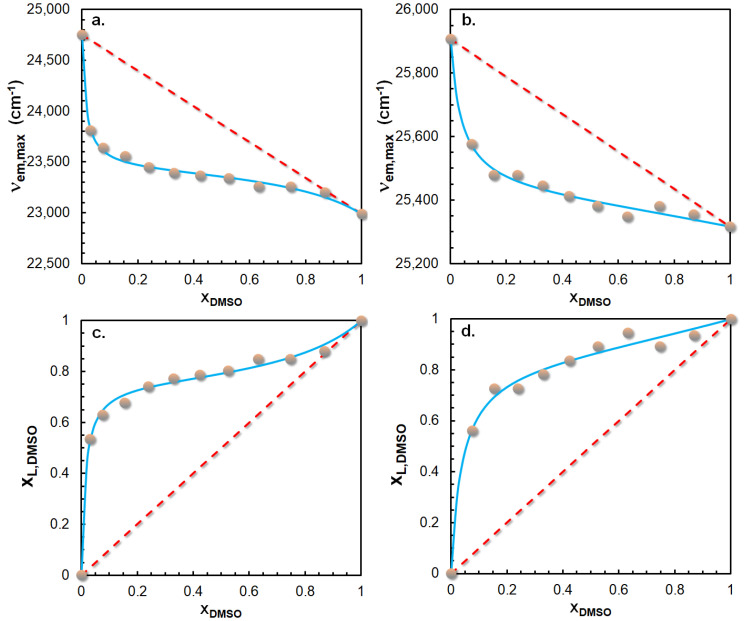
Variations in the emission maxima with the molar fraction of dimethyl sulfoxide (DMSO) in the mixtures of acetonitrile (ACN) and DMSO for (**a**) isocyanonaphthalene-1-ol (ICOL) and (**b**) 5-isocyano-1-(octyloxy)naphthalene (ICON). The change in the local molar fraction of DMSO as a function of the bulk DMSO molar fraction for the (**c**) ICOL and (**d**) ICON. The concentrations of ICOL and ICON were 1.68 μM and 2.45 μM, respectively. The excitation wavelengths for the ICOL and ICON solutions were 320 nm and 325 nm, respectively. The solid blue lines represent the fitted curves calculated by Equation (2) (**c**,**d**) and Equation (3) (**a**,**b**). The red dashed lines represent the ideal behavior.

The presence of emissions originating from the anionic form of ICOL, and the redshift of the ICON fluorescence in the polar solvents, raise the possibility of the presence of preferential solvation by the polar solvents. Thus, to confirm this assumption, a series of ICOL and ICON solutions with different compositions of DMSO and acetonitrile were prepared, and their fluorescence properties were recorded. Figure 7a,b show the change in the emission maxima of the ICOL and ICON (ν_em,max_) in mixtures of DMSO and acetonitrile, respectively, as a function of the DMSO molar fraction (x_DMSO_).

Indeed, as it turns out from Figure 7a,b, both the ICOL and ICON reveal preferential solvation towards DMSO (i.e., the obtained ν_em,max_ values differ considerably from those that can be expected based on ideal behavior (red dashed lines)). Preferential solvation exists if the composition of the solvent mixture in the local environment of the fluorophore deviates from that in the bulk [41,43]. The molar fraction of DMSO in the local environment of ICOL and ICON (x_L,DMSO_) can be expressed by Equation (2) [43,44]:

(2) $$x_{L,\mathrm{DMSO}}=\frac{v_{\mathrm{em},\max}-v_{\max1}}{v_{\max2}-v_{\max1}}$$

where ν_max1_ and ν_max2_ are the emission maxima in Solvent 1 (acetonitrile) and Solvent 2 (DMSO), respectively.

As seen in Figure 7c,d, the molar fraction of DMSO in the local environment of both ICOL and ICON differs from its molar fraction in the bulk. For example, in the case of ICOL at x_DMSO_ = 0.03, the calculated local concentration of DMSO is x_L,DMSO_ = 0.53, which is ca. 18-fold that of the bulk. To describe the variations in the emission maxima (ν_em,max_) with the solvent compositions, the two-step solvent-exchange model was applied (Equation (3)):

(3) $$v_{\mathrm{em},\max}=\frac{v_{\max1}{\left(1-x_{2}\right)}^{2}+v_{\max2}f_{2/1}x_{2}^{2}+v_{\max12}f_{12/1}\left(1-x_{2}\right)x_{2}}{{\left(1-x_{2}\right)}^{2}+f_{2/1}x_{2}^{2}+f_{12/1}\left(1-x_{2}\right)x_{2}}$$

where f_2/1_ and f_12/1_ are the extents of solvation by Solvent 2 (DMSO), and Solvent 1 (acetonitrile) and Solvent 2 with respect to Solvent 1, respectively. ν_max12_ is the emission maximum when the solvation shell contains Solvent 1 (acetonitrile) and Solvent 2 (DMSO) molecules in the same ratios.

Fitting Equation (3) to the ν_em,max_ versus x_DMSO_ curve (Figure 7a, solid blue line) parameters, including ν_max12_, f_2/1_ and f_12/1_, can be determined, for which ν_max12_ = (23,430 ± 30) cm^−1^, f_2/1_ = 19.0 ± 7.7 and 72.9 ± 11.5 were obtained. In contrast to ICOL, it was found that the one-step solvent-exchange model (in this case f_2/1_ = f_12/1_ in Equation (3)) is sufficient for rendering the experimental ν_em,max_ versus x_DMSO_ curve of ICON, with fitted parameters of _max12_ = (25,460 ± 20) cm^−1^ and f_2/1_ = f_12/1_ = 32.5 ± 8.7. The finding that the two-step solvent-exchange model is necessary to predict the fluorescence properties of the ICOL in the DMSO–acetonitrile solvent mixture is likely due to the stronger acid–base-type interaction of ICOL molecules with the solvent molecules in the excited state.

### 2.4. Time-Resolved Emission Properties of ICOL and ICON Derivatives

**Figure 8:**
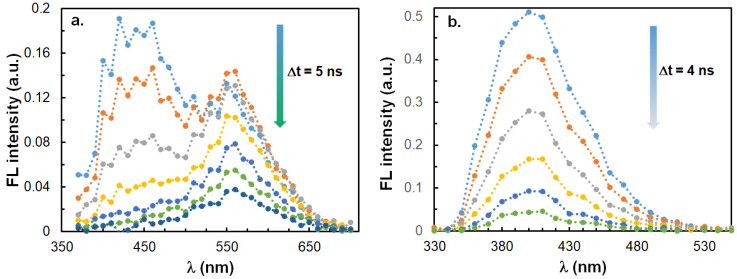
Time-resolved emission spectra for the dimethyl sulfoxide (DMSO) solution of (**a**) 5-isocyanonaphthalene-1-ol (ICOL) and (**b**) 5-isocyano-1-(octyloxy)naphthalene (ICON). The concentrations of ICOL and ICON were 1.77 μM and 1.96 μM, respectively. The excitation wavelength was 355 nm.

The time-resolved emission properties of the ICOL and ICON in six solvents with different polarities were studied by laser flash photometry, and the resulting emission spectra were recorded as a function of time. In Figure 8a,b, the time-resolved emission spectra for the DMSO solutions of ICOL and ICON are shown, respectively.

As seen in Figure 8a, similar to the steady-state fluorescence spectrum of ICOL, its time-resolved emission spectra (TRES) also reveal the presence of two bands at 430 nm and 550 nm, which correspond to the emissions by the neutral isocyanonaphthol molecules and isocyanonaphtholate anions, respectively. Furthermore, it can also be recognized from Figure 8a that the fluorescence intensity of the neutral isocyanonaphthol molecules decreases considerably faster in time than that of the isocyanonaphtholate anions, which brings about an overall redshift in the emissions with time. In addition, the TRES spectra for the DMSO solution of ICON also show a large similarity to its steady-state fluorescence spectrum, which appears as a single band with a maximum wavelength of 395 nm. Conversely, it should be noted that neither the corresponding peak position nor the peak shape of the TRES spectra changed over time. This finding indicates that, after excitation, the solvent molecules had enough time to reorganize themselves around the excited-state fluorophore molecules (i.e., emissions occurred from a solvent-relaxed excited state). The characteristic fluorescence decay rates determined in six different solvents for ICOL and ICON are compiled in Table 5.

**Scheme 1:**
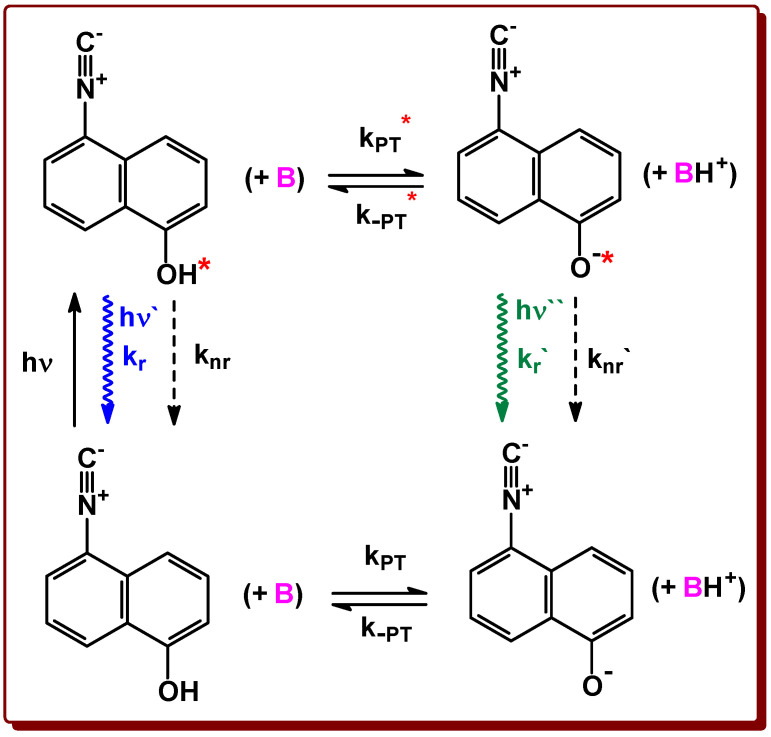
A tentative mechanism proposed for the dual emission of 5-isocyanonaphthalene-1-ol (ICOL). * denotes excited state.

As it turns out from the data in Table 5, the total radiative decay rates for ICON changed only within a narrow range (i.e., from 1.75 × 10^8^ s^−1^ to 2.63 × 10^8^ s^−1^, corresponding to lifetimes from 5.7 ns to 3.8 ns), whereas, for ICOL, these values spanned a wider range (i.e., from 9.9 × 10^7^ s^−1^ to 2.00 × 10^8^ s^−1^, corresponding to lifetimes from 10.1 ns to 5.0 ns), and longer excited-state lifetimes were found for ICOL. Furthermore, it can be surmised from Table 5 that low radiative decay rates for ICOL in polar proton-acceptor solvents, such as DMSO, were determined. The low radiation decay rates may be the consequence of the relatively fast nonradiative deactivation process via hydrogen bonding between the excited-state ICOL and proton-acceptor polar solvent. Based on the experimental results, a tentative mechanism is proposed for the dual emission of ICOL, which is depicted in Scheme 1.

According to Scheme 1, the neutral ICOL is excited from the ground S_o_ state to the S_1_ state. After excitation, in a polar proton-acceptor microenvironment around the fluorophore, two main dominant processes can take place simultaneously: (i) relaxation to the ground state of the neutral ICOL by radiative (k_r_) and nonradiative relaxations (k_nr_), emitting photons of hν’ energy in the former (blue emission); (ii) the excited-state ICOL molecules undergo proton transfer to the microenvironment to yield excited-state isocyanonaphtholate ions (k_PT_*) (i.e., excited-state proton transfer (ESPT) takes place). This latter process is accompanied by the emission of photons of hν’’ energy (green emission), and relaxation to its corresponding ground state (k_r_’), in addition to relaxation by nonradiative decay (k_nr_’). Thus, on the one hand, if k_r_ + k_nr_ > k_PT_*, then partial proton transfer takes place (i.e., emissions are from both the neutral and the anionic forms of ICOL), as observed for, e.g., the DMSO and DMF solutions of ICOL. On the other hand, in the case of k_r_ + k_nr_ < k_PT_*, emissions from only the anionic form can be observed, as is the case for the aqueous solution of ICOL. On the contrary, in microenvironments that are incapable of accepting protons, such as in solvents (e.g., toluene or dichloromethane), the k_PT_* becomes very small compared with k_r_, + k_nr_; thus, only one band due to the neural form appears in the fluorescence spectra.

### 2.5. Fluorescence Properties of ICOL and ICON in the Presence of Surfactants and Bovine Serum Albumin (BSA)

**Figure 9:**
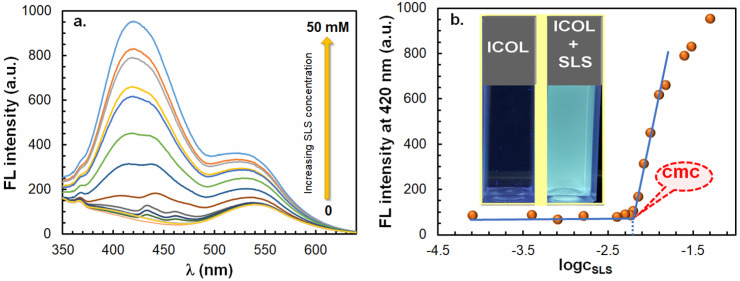
Variations in (**a**) emission spectrum and (**b**) fluorescence intensity at 420 nm for the aqueous solution of 5-isocyanonaphthalene-1-ol (ICOL) in the presence of sodium lauryl sulfate (SLS) of different concentrations. The concentration of ICOL was 50.6 μM. The inset shows the fluorescence of aqueous solution of ICOL in the absence and presence of SLS. The excitation wavelength was 327 nm.

The aqueous solution of ICOL showed only a weak fluorescence with an emission maximum wavelength of 540 nm. However, by adding sodium lauryl sulfate (SLS) to the aqueous ICOL solution in increasing concentrations, the fluorescence became more pronounced, and two additional emission bands at around 415 nm and 442 nm appeared, as shown in Figure 9a. In contrast, since the ICON in aqueous solution revealed a similar quantum-yield value to those in the other solvents (see Table 2), only small changes in the resulting emission intensities could be observed (Figure S16). Thus, in this study, we focused our attention on the fluorescence properties of the aqueous ICOL solution.

Above an SLS concentration of about 6 mM, blue shifts and a considerable intensity increase with increasing SLS concentrations can be observed for all emission bands (Figure 9a). The sudden increase in the fluorescence intensity can be attributed to the formation of micelles with ICOL molecules that reside at the water–micelle interface and/or with ICOL molecules embedded in these micelles. The enhanced fluorescence of ICOL in the presence of SLS is due, most likely, to a reduced availability of water molecules towards ICOL molecules.

It is also evident that, under these circumstances, considerable deprotonation of the ICOL in the excited state (i.e., excited-state proton transfer (ESPT)) takes place to yield the fluorescent isocyanonaphtholate ion. Hence, the presence of ESPT dictates that ICOL molecules reside in a microenvironment that is capable of accepting protons. Furthermore, it is also seen from Figure 9a that, due to the solvatochromic property of ICOL, the emission spectra exhibit the presence of two populations of ICOL molecules: one that resides in a more polar environment (442 nm), and the other that is surrounded by a less polar environment (415 nm). In addition, as the concentration of SLS is increased, the position of the emission maxima shifts to lower emission wavelengths (i.e., from 434 nm to 415 nm and from 462 nm to 442 nm). At higher SLS concentrations, however, these two bands are overlapped, which results in one dominant emission maximum in the range of 400-450 nm. This finding may indicate that a rearrangement of the micelles takes place by increasing the SLS concentration.

As also demonstrated in Figure 9b, the micellization of SLS starts at a critical micelle concentration (*cmc*) of 6.3 × 10^−3^ M (log(c_SLS_) = −2.2), which is close to the reported *cmc* value of SLS in water (8 × 10^−3^ M) [45], which indicates that ICOL is a suitable fluorophore not only for monitoring the *cmc*, but also for detecting the polarity of its microenvironment. From the emission spectra (Figure 9a), it can thus be concluded that ICOL molecules are partially located in the polar microenvironment in the “wetty shell” of the micelle (i.e., at the Stern layer and/or the diffuse Gouy–Chapman layer). This conclusion was further supported by quenching experiments, as will be shown later.

To describe the variation in the fluorescence intensity (F) with the micelle volume to the total volume of the solution (α_m_) (Equation (S28)), the equation presented in [46] was extended and derived for cases when the emission from the solution of free fluorophore, in addition to that of the doped micelles, are also taken into account, as shown by Equation (4). (The derivation of Equation (4) can be found in the Supplementary Material).

(4) $$F=\frac{F_{\infty }}{\alpha_{m}+\frac{1-\alpha_{m}}{P_{m}}}\left[\alpha_{m}+\frac{\beta }{P_{m}}\left(1-\alpha_{m}\right)\right]$$

where P_m_ (Equation (S27)) is the partition coefficient of the fluorophore molecules into the micelles. F_∞_ and β are the fluorescence intensity at α_m_ = 1 and the relative emission-intensity ratio of the aqueous solution of the fluorophore to that of being in the micelle of the same fluorophore concentration, respectively.

To determine the values of P_m_ and β, F values were plotted as a function of the α_m_, and Equation (4) was fitted to the experimental data (Figure S17). For the P_m_ and β, values of 680 + 90 and 0.06 ± 0.02 were obtained, respectively. Similar experiments were also performed in the presence of the nonionic Tween 80 (TW80) surfactant. The successive addition of TW80 to the aqueous solution of the ICOL solution resulted in the emergence of emission bands at 405 nm and 440 nm, in addition to the band at 535 nm that originated from the isocyanonaphtholate ion (Figure S18). The two bands at lower wavelengths, similar to the ICOL-doped SLS micelles, most likely originated from the emissions of ICOL molecules that reside in two microenvironments of different polarities. By plotting the fluorescence intensities as a function of log(c_TW80_), the cmc value of TW80 was also determined, for which, in line with the value reported for TW80 in the literature (0.016 mM), a value of 0.011 mM was obtained (Figure S18) [44]. The P_m_ and β values for the ICOL–TW80 micellar system using Equation (4) were determined to be 4940 ± 2630 and 0.13 ± 0.06, respectively (Figure S19). Although the determined P_m_ and β values show relatively large errors, the P_m_ value is in the same order as that reported for the pyrene-doped Tween 80 micellar system (P_m_ = 4160) [46].

**Figure 10:**
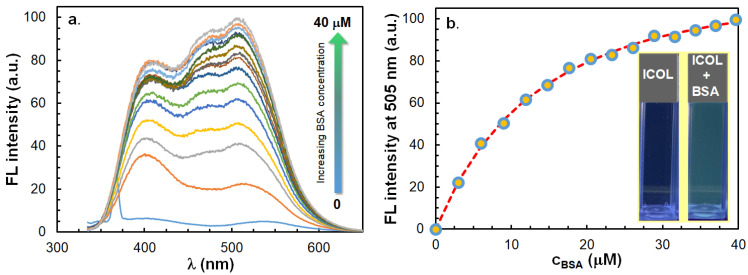
Variation in (**a**) emission spectrum and (**b**) fluorescence intensity at 505 nm for the aqueous solution of 5-isocyanonaphthalene-1-ol in the presence of bovine serum albumin (BSA) at different concentrations. The concentration of 5-isocyanonaphthalene-1-ol (ICOL) was 1.68 μM, and the excitation wavelength was 327 nm. The dashed line stands for the fitted curve by Equation (S36). The inset shows the fluorescence of aqueous solution of ICOL in the absence and presence of BSA.

The enhanced fluorescence properties of ICOL when partitioning it from water into a less polar environment can also be utilized (e.g., its partition into biomolecules, such as bovine serum albumin (BSA)). Indeed, as Figure 10a and the Figure 10b inset show, the emission intensities at both 405 nm and 505 nm increased upon the addition of BSA, which indicates the elevated concentration of BSA–ICOL associates. It is to be noted that the solution of BSA excited at 327 nm revealed a blue fluorescence, with a maximum wavelength of 414 nm; thus, the emission spectra of the BSA solutions of different concentrations were subtracted from the corresponding emission spectra of the BSA–ICOL solutions. The increased fluorescence of ICOL in the presence of BSA can be attributed to a decreased exposition of water molecules towards ICOL molecules embedded in a less polar region of BSA. Furthermore, it is also evident that, under these conditions, the significant deprotonation of ICOL takes place in the excited state. However, the resulting ESPT band is most likely due to a reduced ability of the excited-state anion buried in a less polar microenvironment of BSA to form hydrogen bonds, as compared with bulk water. In addition, upon increasing the BSA concentration, a new band at around 475 nm appeared. The presence of the additional band in the emission spectra may indicate that ICOL molecules can bind to cavities of different polarities of BSA. Due to the excess of BSA to ICOL employed in our experiments, it can be surmised that ICOL–BSA associates with a composition of 1:1 (ICOL/BSA) were formed, and emissions at 505 nm were further selected to monitor the formation of ICOL–BSA associates, as demonstrated in Figure 10b.

On the one hand, as seen in Figure 10b, the addition of BSA in increasing concentrations resulted in a subsequent enhancement of the emissions at 505 nm, up to a limit value. On the other hand, the variation in the fluorescence intensity at 505 nm can adequately be described by Equation (S38), as judged from its fitting to the experimental data. The details of the model can be found in the Supplementary Material. From the fitting (Equation (S38)), the apparent association constant (K_AS_) between the BSA and ICOL was found to be (6.7 ± 0.3) × 10^4^ M^−1^.

### 2.6. Fluorescence Quenching by Pyridine

**Figure 11:**
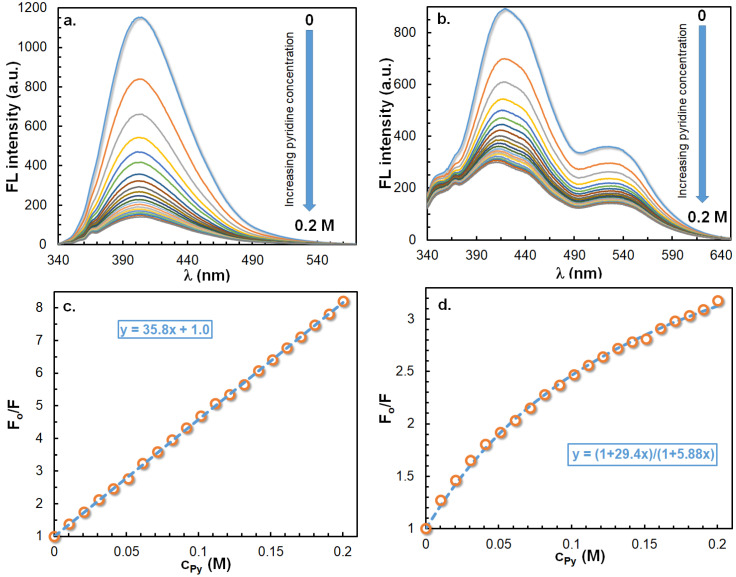
Variations in the emission spectra with the pyridine concentration (Py) for the (**a**) acetonitrile solution of 5-isocyanonaphthalene-1-ol (ICOL) and (**b**) aqueous solution of ICOL in the presence of sodium lauryl sulfate (SLS) at a concentration of 25 mM. The concentration of ICOL was 1.2 × 10^−4^ M, and the excitation wavelength was 327 nm. (**c**,**d**) demonstrate the Stern–Volmer plots for Figure 11a at 404 nm and Figure 11b at 430 nm, respectively. The blue dashed lines are the fitted curves by Equations (5) and (6).

During the study of the solvatochromic properties of the ICOL and ICON derivatives, it was found that the pyridine solutions of ICOL and ICON showed no fluorescence at all, which indicates the quenching of emissions by pyridine. In order to investigate the mechanism of the quenching of emissions, acetonitrile solutions of pyridine in different concentrations were added to the acetonitrile solutions of ICOL and ICON, and their emissions were recorded. Figure 11a shows the variation in the steady-state emission spectra upon the addition of pyridine in increasing concentrations to the solution of ICOL, while Figure 11c depicts the Stern–Volmer plot (i.e., the values of the F_o_/F ratio as a function of the pyridine concentration (Py)), where F_o_ and F are the initial and instant fluorescence intensities at 404 nm, respectively.

(5) $$\frac{F_{o}}{F}=1+K_{a}c_{\mathrm{Py}}$$

where K_a_ is the quenching constant, and c_Py_ stands for the concentration of pyridine.

As seen in Figure 11a, upon increasing the pyridine concentration, the intensities of emissions subsequently decreased, without any shift in the emission maximum. Furthermore, from the slope of the Stern–Volmer plot, a K_a_ value of 35.8 ± 0.2 was obtained for ICOL, while, for ICON under the same conditions, a similar value of K_a_ = 34.1 ± 0.2 was determined (Figure S20). To learn whether the quenching process is dynamic or static in nature, time-resolved emission spectra (TRES) were also recorded for the acetonitrile solution of ICOL. The results of the TRES experiments for the ICOL revealed that the excited-state lifetime decreased from a τ = 8.1 ns to 3.5 ns upon increasing the concentration of pyridine from 0 to 0.1 M. In addition, plotting the value of τ_o_/τ as a function of the pyridine concentration also yielded a straight line with a slope of 36.6 ± 2.6 (Figure S21), which is very close to the value determined for the ICOL by the steady-state fluorescence method. This finding clearly and unambiguously indicates that quenching by pyridine is a pure dynamic process.

It was also intriguing to investigate the quenching of ICOL by pyridine in the presence of SLS and TW80 micelles above the cmc. As expected, according to Figure 11b, the addition of pyridine to the micellar solution resulted in subsequent decreases in the fluorescence intensities, without any shift in the emission spectra. Moreover, the Stern–Volmer plot (Figure 11d), in contrast to the micelle-free solution of ICOL (Figure 11c), reveals a downward curvature, which suggests the presence of two ICOL populations: one of which is not, or is less, accessible to the quencher pyridine molecules, while the other fraction is fully available for quenching. The inaccessible fraction of ICOL molecules to quencher molecules (f_na_) can be determined by the modified Stern–Volmer equation (Equation (6) and Figure 11d) [4]:

(6) $$\frac{F_{o}}{F}=\frac{1+K_{a}[\mathrm{Py}]}{1+f_{\mathrm{na}}K_{a}[\mathrm{Py}]}$$

By fitting Equation (6) to the experimental F_o_/F data, the values of the K_a_ and f_na_ can be estimated, for which 29.4 ± 0.7 and 0.20 ± 0.01 were obtained, respectively. Based on these data, it can be surmised that the value of the quenching constant is very similar to that obtained in the absence of micelles (in acetonitrile). It can further be concluded that ca. 4/5 of the ICOL molecules are located near the polar surface (i.e., at the Stern and/or Gouy–Chapman layers), whereas ca. 1/5 of the fluorophore molecules are buried in the nonpolar region of the SLS micelles, which makes these molecules inaccessible to the quencher molecules. Similar quenching experiments were performed for ICOL-doped TW80 micelles. The Stern–Volmer plot for the ICOL–TW80 micellar system also showed a downward curvature, similar to that found for the ICOL–SLS system (Figure S22), which also indicates the presence of a nonavailable fraction of ICOL molecules for the quencher pyridine molecules. As a result of the fitting of Equation (6) to the experimental data, K_a_ = 21.0 ± 1.0 and f_na_ = 0.33 ± 0.01 were obtained. The obtained quenching constant and f_na_ values for the ICOL–TW80 micelles indicate that ICOL molecules are less accessible to the quencher pyridine molecules. Thus, the quenching experiments by pyridine allows for the determination of the ratio of the populations of ICOL molecules residing in the interfacial and interior spaces of supramolecular assemblies.

## 3. Experimental

### 3.1. Chemicals

5-amino-1-naphthol, phosphorous oxychloride, octylbromide, formic acid, sodium lauryl sulfate (SLS), Tween 80 (TW80) and bovine serum albumin (BSA) were purchased from Sigma–Aldrich (Darmstadt, Germany) and used without further purification. Dichloromethane, 2-propanol chloroform, ethyl acetate and toluene, from Molar Chemicals (Budapest, Hungary), were purified by distillation. Acetonitrile, tetrahydrofuran, methanol, dimethylformamide, dimethyl sulfoxide (DMSO), pyridine (HPLC grade, VWR, Darmstadt, Germany) and 1,4-dioxane (reagent grade) were received from Reanal (Budapest, Hungary).

### 3.2. Synthesis of 5-isocyanonaphthalene-1-ol (ICOL) and 5-isocyano-1-(octyloxy)naphthalene (ICON)

**Scheme 2:**
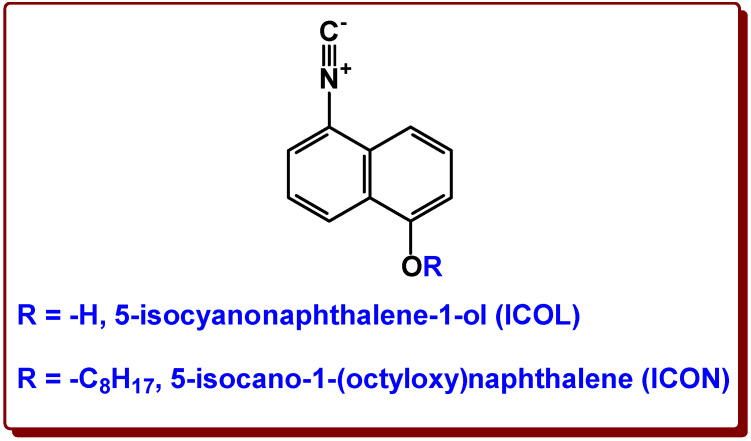
The chemical structure of the investigated compounds.

The chemical structures of the synthesized compounds are shown in Scheme 2. The synthesis procedure and ^1^H-NMR, ^13^C-NMR (nuclear magnetic resonance) and mass spectrometric characterization for the ICOL (Figures S1–S3) and ICON (Figures S4–S6) derivatives are described in the Supplementary Materials.

### 3.3. Methods and Instrumentation

The electronic absorption spectra (UV–Vis) were recorded on an Agilent Cary 60 spectrophotometer (Agilent Technologies, Inc., Santa Clara, CA, USA) in a quartz cuvette with a 1 cm optical length.

Fluorescence measurements were carried out using a Jasco FP-8500 fluorescence spectrophotometer (JASCO Corporation, Tokyo, Japan) equipped with a Xe-lamp light source. The excitation and emission spectra were recorded at 25 °C using 2.5 nm excitation, a 5.0 nm emission bandwidth and a 50–200 nm/min scan speed. Fluorescence quantum yields were determined by using quinine-sulfate in 0.1 mol/L sulfuric acid as the reference (absolute quantum efficiency: Q_F_ = 0.55).

For UV–Vis measurements, ICOL was dissolved in the corresponding solvents at a concentration of 8.42 mM, and diluted to obtain concentrations from 34.2 μM to 168 μM, whereas ICON, dissolved at a concentration of 5.75 mM, was diluted to obtain concentrations from 23.5 μM to 115 μM.

For fluorescence measurements, ICOL was dissolved in the corresponding solvents at a concentration of 0.34 mM and diluted to 1.68 μM, while ICON, dissolved at a concentration of 0.49 mM, was diluted to attain 2.46 μM.

Solutions for determining the critical micelle concentration (CMC) of SLS and TW80 were prepared by mixing aqueous solutions of 2955 μL of SLS or 2955 μL of TW80 with 45 μL of ICOL or ICON dissolved in acetonitrile. The final concentrations of the ICOL and ICON were 50.6 μM and 7.4 μM, respectively, whereas those of the SLS and TW80 were in the range of 0.8 mM–50 mM and 0.16 μM–100 μM, respectively. Solutions for the fluorescence-quenching experiments by pyridine were prepared in acetonitrile or water in the presence of SLS or TW80 micelles at concentrations of 25.1 mM and 50.0 μM, respectively, and at an ICOL concentration of 50.6 μM. The fluorescence of ICOL solutions in the BSA in phosphate-buffered-saline (PBS) solution (pH = 7.4) was studied using an ICOL concentration of 1.68 μM and BSA concentrations up to 40 μM. The UV–Vis measurements for the determination of the ground-state pK_a_ value of ICOL were performed at an ICOL concentrations of 19.7 μM in Britton–Robinson buffer (BRB) in the range of pH = 5–11, while the excited-state pK_a_ value (pK_a_*) was estimated in HCl solutions in a concentration range from 1mM to 1 M. Preferential solvation of ICOL and ICON towards DMSO were investigated in the mixtures of the acetonitrile and DMSO of different compositions, at ICOL and ICON concentrations of 1.68 μM and 2.45 μM, respectively.

Laser-flash-photolysis experiments were carried out with an Applied Photophysics LKS.60 nanosecond transient absorption spectrometer (Applied Photophysics Ltd., Leatherhead, UK), equipped with a Quantel Brilliant Nd:YAG laser (Lumibird, Paris, France), along with its second, third and fourth harmonic generators. The third harmonic, emitting at 355 nm, was used. The solutions for time-resolved emissions (TRES) were prepared in acetonitrile, toluene, tetrahydrofuran, dichloromethane, methanol and dimethyl sulfoxide at ICOL and ICON concentrations of 1.77 μM and 1.96 μM, respectively. For the evaluations of the time-resolved emission data, “DecayFit 1.4” software, downloaded freely from www.fluortools.com (accessed on 1 February 2022), was used with the tail-fitting method to determine the corresponding fluorescence decay rates.

### 3.4. Time-Dependent Density-Functional-Theory (TD-DFT) Calculations

The density-functional-theory (DFT) calculations were carried out using the Gaussian16 software package [47]. To obtain the ground-state properties, the ICOL and ICON molecules were optimized in the polarizable-continuum-model (PCM) [48] solvent (toluene and DMSO) cavity using the M06 functional [49] together with the TZVP basis set [50]. The octyl group of ICON was replaced with a methyl group to remove its conformational complexity. This truncation has no effect on the electronic spectra. Frequency calculations were performed on the optimized structures to verify them as stationary points and to obtain Gibbs-free-energy corrections. The vertical excitations and excited-state geometries (S_1_ relaxation) were calculated using time-dependent density functional theory (TD-DFT), employing the M06 functional and the TZVP basis set, together with linear-response solvation through the PCM approach [51]. In addition to modeling the vertical excitation, the molecules were optimized on the potential surface of the first singlet excited state to obtain the excited-state properties, such as the dipole moments and electrostatic potentials. For the absorption and emission, the first 30 and 20 excited states were calculated, respectively. The solvent parameters were set to their default values, and the cavity volumes of the optimized ground- and excited-state geometries are collected in Table S3. The continuous spectra were obtained through the Gaussian fitting and normalization procedure described in [51], using a width parameter of 0.14 eV. To analyze the charge-transfer (CT) behavior of the ICOL, the Δr and Λ parameters were calculated alongside the electron–hole analysis using the Multiwfn software [52]. The CAM-B3LYP [42] functional was also tested for the TD-DFT and CT analyses. It severely underestimates the emission wavelengths (Figure S8) and offers less accurate absorption wavelengths compared with M06, as shown in Table S4. Both functionals predicted similar CT behavior for the first excited state, as shown in Figure S11, Figure 5 and Table S6. Because the obtained Δr values near 1.5 Å indicate a short-range CT interaction [52], the M06 functional is expected to provide reasonable results, and no long-range corrections are necessary for the molecules in this work.

## 4. Conclusions

The synthesized ICOL and ICON derivatives showed a positive solvatochromic effect, with moderate Stokes shifts in the range from 5208 cm^−1^ to 7407 cm^−1^ for ICOL, and from 3595 cm^−1^ to 5675 cm^−1^ for ICON, in solvents from nonpolar to polar ones. The difference in the dipole moments of the ground and excited states, as well as those extracted from the fitting procedure for the ground and excited states separately, using various solvent polarity functions, were found to agree well with the results obtained by the TD-DFT. The solvatochromic shifts of the ICOL and ICON were also interpreted in terms of the Kamlet–Taft method. Furthermore, dual fluorescence with blue emissions attributable to the neutral form of ICOL, and green emissions due to the anionic deprotonated form, were observed in solvents of high polarity and capable of accepting protons. ICON, however, due to the lack of mobile protons, did not exhibit dual emission. The ground- and excited-state pK_a_ values of the ICOL were determined to be 8.4 ± 0.3 and 0.9 ± 0.7, respectively. The excited-state pK_a_ values are in line with those calculated by the Förster cycle (0.07). The considerable decrease in the pK_a_ for the excited state makes ICOL suitable as a photoacid, while its solvatochromic property allows one to screen the polarity of the microenvironment around the fluorophore. Furthermore, it was also pointed out that both ICOL and ICON revealed preferential solvation towards DMSO, and ICOL can dynamically be quenched by pyridine. It was also shown that ICOL, in contrast to ICON, revealed only very weak fluorescence in aqueous solution. However, in the presence of surfactants at concentrations above the cmc, the emissions were enhanced, which allowed for the determination of the cmc. The determined cmc values for the SLS and TW80 were found to be in good agreement with those reported in the literature. In addition, it was concluded from the emission spectra of the ICOL, recorded in the presence of SLS or TW80, that a certain fraction of ICOL molecules most likely reside at the water–micelle interface, while the rest are located inside the micelles, as was also confirmed by the pyridine-quenching experiments. Similarly, in the presence of BSA, ICOL also exhibited enhanced fluorescence with the occurrence of additional bands, which indicated different microenvironments around the ICOL molecules. As a conclusion, it was pointed out that ICOL is a very promising “dual” fluorophore that carries both solvatochromic and ESPT features for exploring the polarity and proton-acceptor capability of the microenvironment at the interface and/or in the interior spaces of various assemblies, such as micelles, vesicles and biomacromolecules. ICOL, due to its relatively low-excited-state pK_a_^*^ value, may be applied as a photoacid for biomedical applications as well. Furthermore, ICOL can be expected to form complexes predominantly through its isocyano group with transition-metal ions, such as Cu^2+^, Ag^+^ and Au^+^, or can potentially be anchored to the surfaces of various nanoparticles (e.g., silver or gold) to enhance their optical properties and/or photoacidity.

## Figures and Tables

**Table 1:** The optical properties of 5-isocyanonaphthalene-1-ol (ICOL) dissolved in different solvents.

5-isocyanonaphthalene-1-ol						
No.	Solvent	λ_em_ (nm)	λ_ex_ (nm)	Δν (cm^−1^)	*ε* (M^−1^cm^−1^)	Q_F_
1	Toluene	384	320	5208	6005	0.44
2	Dioxane	389	323	5253	5540	0.60
3	Ethyl acetate	392	322	5546	6420	0.43
4	Dichloromethane	395	318	6130	6150	0.70
5	Tetrahydrofuran	395	329	5079	6160	0.69
6	Chloroform	402	322	6180	5510	0.51
7	Acetonitrile	404	320	6498	5900	0.47
8	2-propanol	410	330	5913	5330	0.12
9	Methanol	415	329	6299	6220	0.04
10	*N*,*N*-dimethyformamide	432	330	7155	6200	0.07
11	Dimethyl sulfoxide	435	329	7407	5200	0.05

λ_em_, λ_ex_, Δν, ε and Q_F_ stand for the emission maximum, excitation maximum (absorbance maximum), Stokes shift, molar absorption coefficient at λ_ex_ and quantum yield, respectively.

**Table 2:** The optical properties of 5-isocyano-1-(octyloxy)naphthalene (ICON) dissolved in different solvents.

5-isocyano-1-(octyloxy)naphthalene						
No.	Solvent	λ_em_ (nm)	λ_ex_ (nm)	Δν (cm^−1^)	*ε* (M^−1^cm^−1^)	Q_F_
1	Toluene	368	325	3595	10,890	0.36
2	Dioxane	375	326	4008	11,900	0.50
3	Ethyl acetate	376	325	4173	11,920	0.33
4	Dichloromethane	374	320	4512	11,190	0.17
5	Tetrahydrofuran	377	327	4056	12,020	0.41
6	Chloroform	385	328	4514	10,870	0.07
7	Acetonitrile	385	325	4795	10,850	0.38
8	2-propanol	394	322	5675	10,020	0.42
9	Methanol	395	320	5934	9690	0.33
10	*N*,*N*-dimethyformamide	387	330	4463	11,800	0.50
11	Dimethyl sulfoxide	395	334	4624	11,820	0.68

λ_em_, λ_ex_, Δν, ε and Q_F_ stand for the emission maximum, excitation maximum (absorbance maximum), Stokes shift, molar absorption coefficient at λ_ex_ and quantum yield, respectively.

**Table 3:** The ground-state (μ_G_), excited-state (μ_E_) and difference between excited- and ground-state (Δμ) dipole moments determined by the Weller (W), Lippert–Mataga (LM), Bilot–Kawski (BK), McRea (MR), Reichardt (R) and time-dependent density functional theory (TD-DFT) methods for the 5-isocyanonaphthalene-1-ol and the 5-isocyano-1-(octyloxy)naphthalene. The dipole moments are given in Debye (D) units.

	5-isocyanonaphthalene-1-ol (ICOL)					
	W	LM	BK	MR	R	TD-DFT
**μ_G_ (D)**	-	-	5.6 ± 0.4	-	-	5.3
**μ_E_ (D)**	6.9 ± 1.0	-	9.0 ± 0.5	-	-	9.3
Δ**μ (D)**	-	5.5 ± 1.1	3.4 ± 0.9	3.7 ± 0.6	3.4 ± 0.3	4.0
	**5-isocyano-1-(octyloxy)naphthalene (ICON)**					
**μ_G_ (D)**	-	-	2.5 ± 0.4	-	-	5.8
**μ_E_ (D)**	5.6 ± 0.8	-	5.7 ± 0.5	-	-	10.4
Δ**μ (D)**	-	5.6 ± 0.9	3.2 ± 0.9	3.6 ± 0.6	2.3 ± 0.2	4.6

**Table 4:** The Kamlet–Taft parameters (Y_o_, a_α_, b_β_, c_π*_) and their errors for the 5-isocyanonaphthalene-1-ol and the 5-isocyano-1-(octyloxy)naphthalene estimated from the emission maximum (ν_f_) and Stokes shift (Δν).

	5-isocyanonaphthalene-1-ol (ICOL)			
	Y_o_ (cm^−1^)	a_α_ (cm^−1^)	b_β_ (cm^−1^)	c_π*_ (cm^−1^)
**ν_f_ (cm^−1^)**	28,320 ± 2890	−615 ± 245	−1730 ± 310	−4180 ± 445
Δ**ν (cm^−1^)**	3250 ± 380	555 ± 320	315 ± 210	4000 ± 580
	**5-isocyano-1-(octyloxy)naphthalene (ICON)**			
**ν_f_ (cm^−1^)**	28,100 ± 190	−1050 ± 130	−1305 ± 235	−1735 ± 250
Δ**ν (cm^−1^)**	3730 ± 1020	2520 ± 700	−2540 ± 1270	2980 ± 1360

**Table 5:** Radiative (k_r_), nonradiative (k_nr_) and total fluorescence decay rates (k_t_) for 5-isocyanonaphthalene-1-ol and 5-isocyano-1-(octyloxy)naphthalene measured in different solvents. The determinations of k values are within ± 10 % error.

	Fluorescence Decay Rates (k_t_, k_r_, k_nr_) × 10^−8^ (s^−1^)					
	*5-isocyanonaphthalene-1-ol*			*5-isocyano-1-(octyloxy)naphthalene*		
	k_t_	k_r_	k_nr_	k_t_	k_r_	k_nr_
Toluene	1.41	0.62	0.79	2.44	0.88	1.56
Tetrahydrofuran	1.25	0.86	0.39	2.63	1.08	1.55
Dichloromethane	0.99	0.69	0.30	1.96	0.33	1.63
Acetonitrile	1.23	0.58	0.65	2.33	0.89	1.44
Methanol	2.00	0.08	1.92	1.96	0.65	1.31
Dimethyl sulfoxide	1.23 *, 0.68 **	(0.03) (0.02)	(1.2) (0.66)	1.75	1.19	0.56

* Determined at 440 nm. ** Determined at 550 nm.

## Data Availability

Additional data are available from the authors upon request.

## Supplementary Materials

Supplementary materials can be found at https://www.mdpi.com/article/10.3390/ijms23137250/s1.
