# Supplementary material for: Isocyanonaphthol Derivatives: Excited-State Proton Transfer and Solvatochromic Properties
Source: Int J Mol Sci. 2022 Jun 29;23(13):7250. doi: 10.3390/ijms23137250 (PMC9266744; doi:10.3390/ijms23137250)
Supplement: Supplementary file 1 [file ijms-23-07250-s001.zip › ijms-1772197-supplementary.pdf]

Supplementary Material for

Isocyanonaphthol derivatives: excited-state proton transfer and solvatochromic properties

Anita Adamoczky[1,2], Tibor Nagy[1], Péter Pál Fehér[3], Veronika Pardi-Tóth[1], Ákos Kuki[1], Lajos Nagy[1], Miklós Zsuga[1], Sándor Kéki[1*]

[1]Department of Applied Chemistry, Faculty of Sciences and Technology, University of Debrecen, Egyetem tér 1, H-4032 Debrecen, Hungary;
[2]Doctoral School of Chemistry, University of Debrecen, Egyetem tér 1., H-4032 Debrecen, Hungary
[3]Research Centre for Natural Sciences, Magyar tudósok körútja 2., H-1117 Budapest, Hungary

[*]Correspondence: keki.sandor@science.unideb.hu (S.K.); Tel: +36-52-512-900 (ext. 22455)

Table of Contents

_Toc107054707

1. Synthesis and characterization of 5-isocyanonaphthalene-1-ol and 5-isocyano-1-(octyloxy)naphthalene 2

1.1. Synthesis and characterization of 5-isocyanonaphthalene-1-ol (ICOL) (and Fig. S1-Fig. S3) 2

1.2 Synthesis and characterization of 5-isocyano-1-(octyloxy)naphthalene (ICON) (and Fig. S4-Fig. S6) 6

2. Evaluation of the solvatochromic properties of ICOL and ICON 10

2.1. The Weller method 10

2.2. The Lippert-Mataga method 10

2.3. The McRea method 11

2.4. The Bilot-Kawski method 11

2.5. The Reichardt method 12

2.6. The refractive index and the permittivity values and the Kamlet-Taft parameters used for the evaluation (and Table S1 and Table S2) 13

3. The results of additional TD-DFT calculations (and Table S3-S7 and Fig. S7-S13) 14

4. Derivation of the expression for the determination of pK_a and the apparent pK_a*values (and Fig. S14 and Fig. S15) 22

5. Derivation of eq. (6) and emission intensities *versus* surfactant concentrations (and Fig. S16-S19) 25

6. Derivation of the determination of the association constant between ICOL and BSA 29

7. Quenching of ICOL and ICON solutions by pyridine (and Fig. S20-Fig. S22) 31

1. Synthesis and characterization of 5-isocyanonaphthalene-1-ol and 5-isocyano-1-(octyloxy)naphthalene

1.1. Synthesis and characterization of 5-isocyanonaphthalene-1-ol (ICOL) (and Fig. S1-Fig. S3)

A 250 ml three-necked round-bottomed flask, equipped with a condenser, dropping funnel, argon inlet-outlet and magnetic stirrer, was charged with the 5-amino-1-naphthol (1.00 g, 6.28 mmol) toluene (84 mL) and formic acid (9.76 mL, 0.25 mol). The reaction mixture was stirred for 3 hours under argon atmosphere at a temperature of 40 ºC followed by addition of dichloromethane of 20 mL to the reaction mixture and stirred for an additional 10 min at room temperature. The toluene-dichloromethane phase was decanted and the solid precipitate formed was dissolved in ethyl acetate of 100 mL then extracted tree times with distilled water of 50 mL. The resulting solution was dried over magnesium sulfate and the solvent was evaporated to yield N-(5-hydroxynaphthalen-1-yl)formamide (730 mg, 3.90 mmol, yield: 66.4 %).

In the second step, a three-necked round-bottomed flask under argon atmosphere over an ice bath was charged with (5-hydroxynaphthalen-1-yl)formamide (730 mg, 3.90 mmol) dichloromethane of 100 mL, 168.6 mg (1.38 mmol) 4-dimethylaminopyridine (DMAP), 1.53 mL (10.9 mmol) trietilamin (TEA) and 0.42 mL (4.29 mmol) $POCl_3$ slowly dripped to the reaction mixture through a septum. The reaction mixture was stirred for 10 min at 0°C and for addition 2.5 hour at room temperature then 45 mL aqueous solution of Na_2CO_3 (10 % m/m) was added and stirred for 10 min. The organic phase was separated and washed tree times with distilled water of 50 mL followed by drying over magnesium sulfate. The solvent was evaporated by a rotary evaporator and the product was purified by normal phase column chromatography using dichloromethane as the eluent to yield crystalline 5-isocyanonaphthalene-1-ol (0.025 g, 0.15 mmol, yield: 3.8 %).

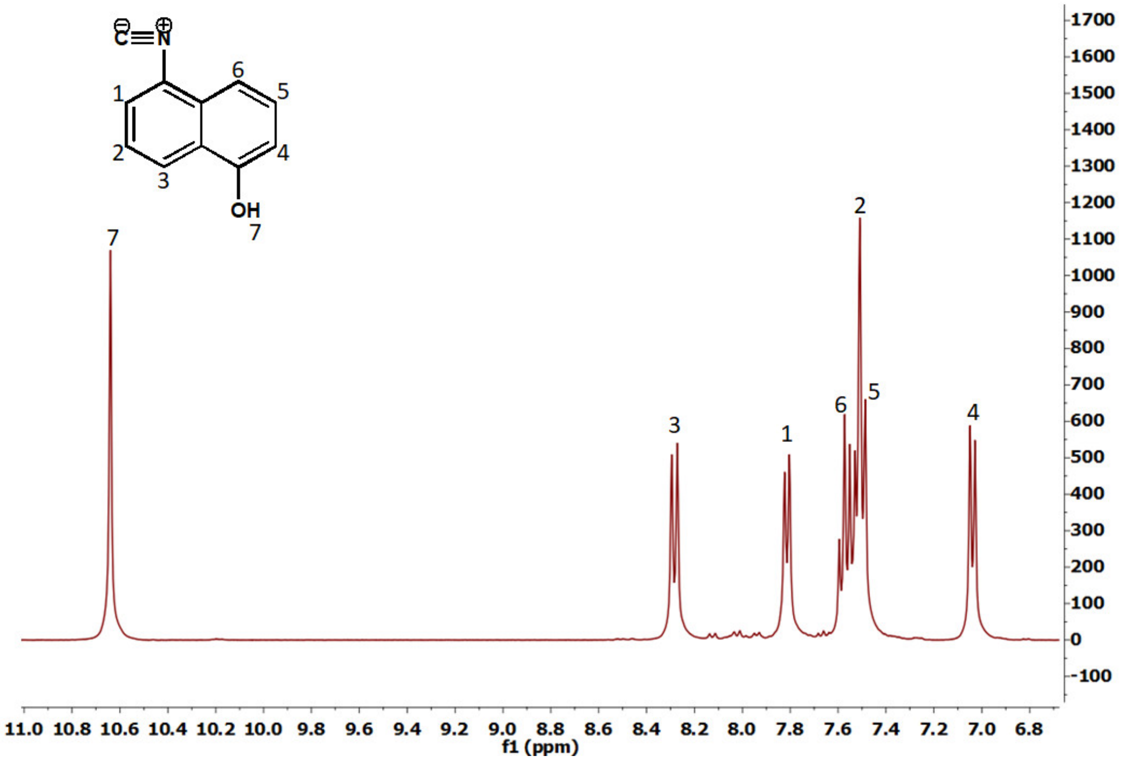

Figure S1. ^{1}H-NMR spectrum of the of 5-isocyanonaphthalene-1-ol (ICOL). Chemical shifts (δ_H), (DMSO-d_6, relative to TMS, 360 MHz): 7.03 ppm (2H), 7.55 ppm (3H), 7.81ppm (1H), 8.28 ppm (1H), 10.64 ppm (1H, C-OH).

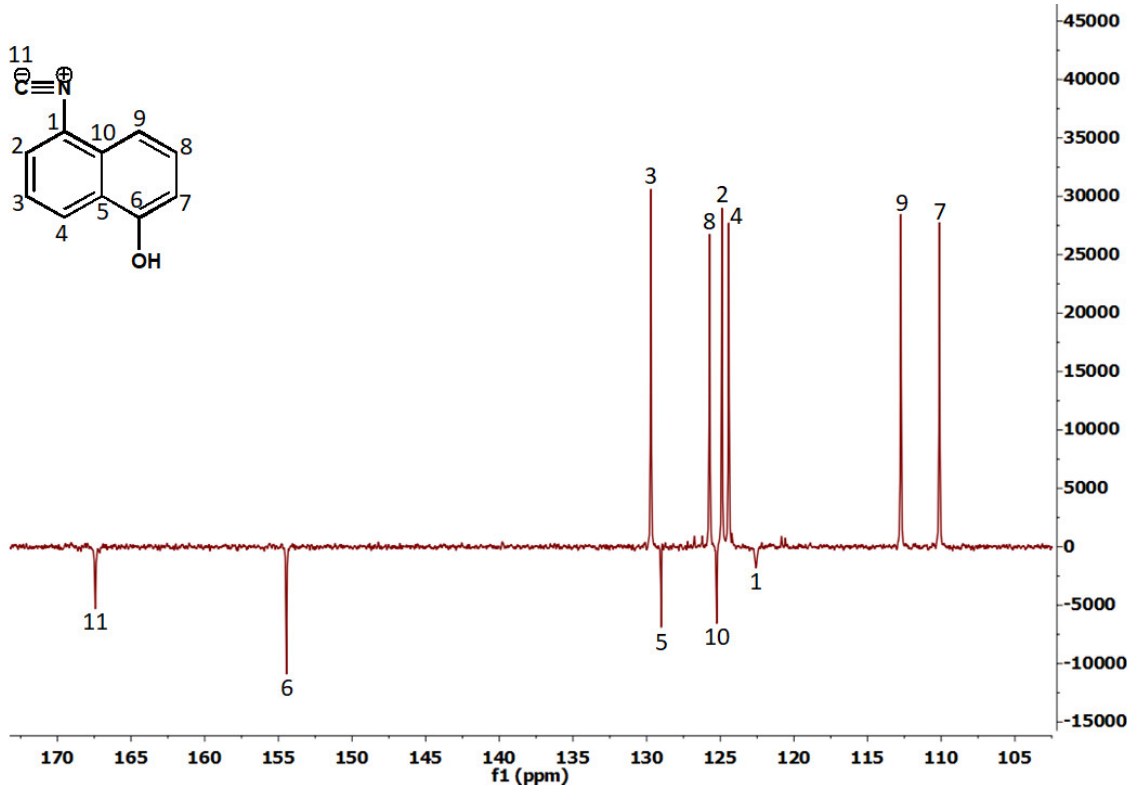

Figure S2. ^{13}C-NMR spectrum of the of 5-isocyanonaphthalene-1-ol (ICOL). Chemical shifts (δ_C), (DMSO-d_6, 90 MHz): 110.0 ppm (C2), 112.7 ppm (C4), 122.5 ppm (C8), 124.4 ppm (C6), 124.8 ppm (C9), 125.2 ppm (C7), 125.7 ppm (C3), 129.0 ppm (C10), 129.7 ppm (C1,C5), 167.4 ppm (NC).

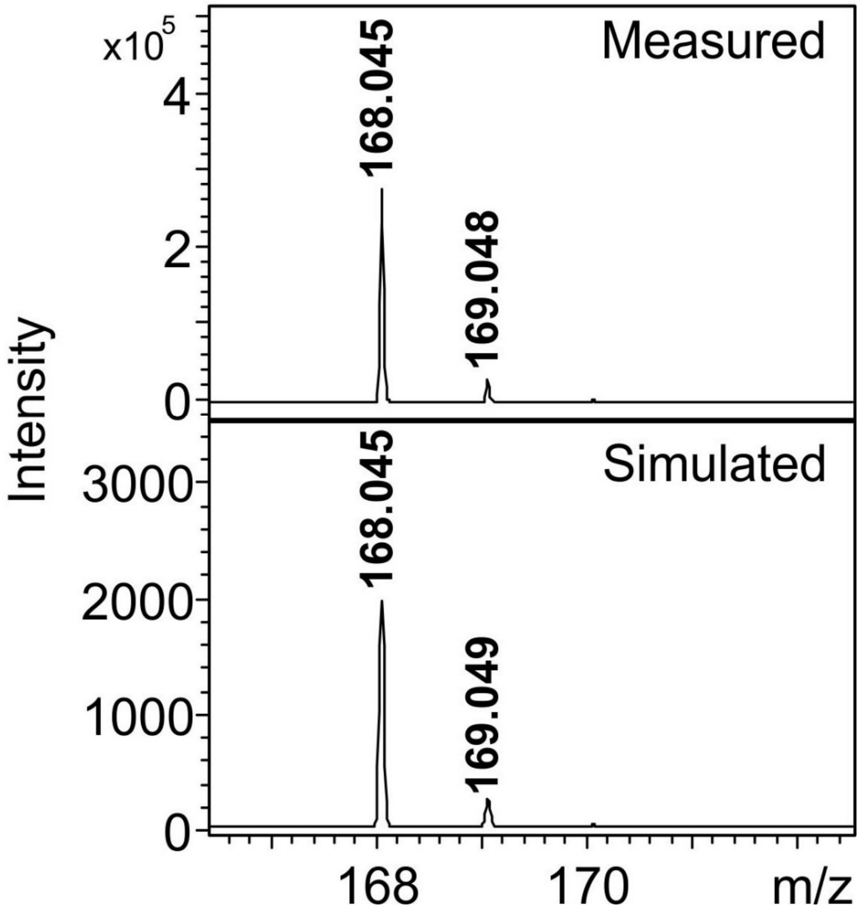

Figure S3. ESI-TOF MS spectrum of the of 5-isocyanonaphthalene-1-ol (ICOL).

1.2 Synthesis and characterization of 5-isocyano-1-(octyloxy)naphthalene (ICON) (and Fig. S4-Fig. S6)

Into a three-necked flask of 50 mL, 1 g (6.28 mmol) 5-amino-1-naphthol and 2 mL 1-propanol, grinded sodium hydroxide (1.26 g, 31.4mmol) and 1-bromo-octane (1.30 mL, 7.54 mmol) were added and the reaction mixture was stirred under argon atmosphere at room temperature for 1 hour then for an additional 23 hours at 70 °C. After cooling to room temperature, ethyl acetate of 6 mL was added to the reaction mixture and extracted with 3 x 10 mL distilled water. After evaporation of the solvent 2.03 g crude oil-like material was obtained.

In the second synthesis step, into a three-necked flask crude product of 2.03 g obtained in the first synthesis step, 168 mL toluene and 19.52 mL (0.5 mol) formic acid were added and stirred for 1.5 hour under argon atmosphere at 80 °C. After cooling the reaction mixture to room temperature, dichloromethane of 80 mL and aqueous solution of $NaHCO_3$ (10 % m/m) of 40 mL was added and the organic phase was washed with distilled water (3x100 mL) followed by drying over $MgSO_4$. After filtration, the solvents were evaporated by a rotary evaporator. The dry product obtained was then dissolved in dichloromethane of 100 mL and transferred into a three-necked flask together with 13.06 mL (0.0937 mol) trimethylamine and 4.44 mL (0.0474 mol) $POCl_3$ was slowly dripped into the reaction mixture that was continuously stirred and kept under argon atmosphere at 0 °C. The reaction was followed by thin-layer chromatography and after 1hour reaction time, 200 mL aqueous Na_2CO_3 (10 % m/m) and 100 mL dichloromethane were added to the reaction mixture. After separation, the organic phase was washed with 3 x 100 mL distilled water, dried over MgSO4 and after filtration, the reaction mixture was evaporated to dry by a rotary evaporator. The product obtained was purified by normal-phase column chromatography using dichloromethane as the eluent to yield 5-isocyano-1-(octyloxy)naphthalene (ICON) (0.20 g, 0.71 mmol, yield: 11.3 %)

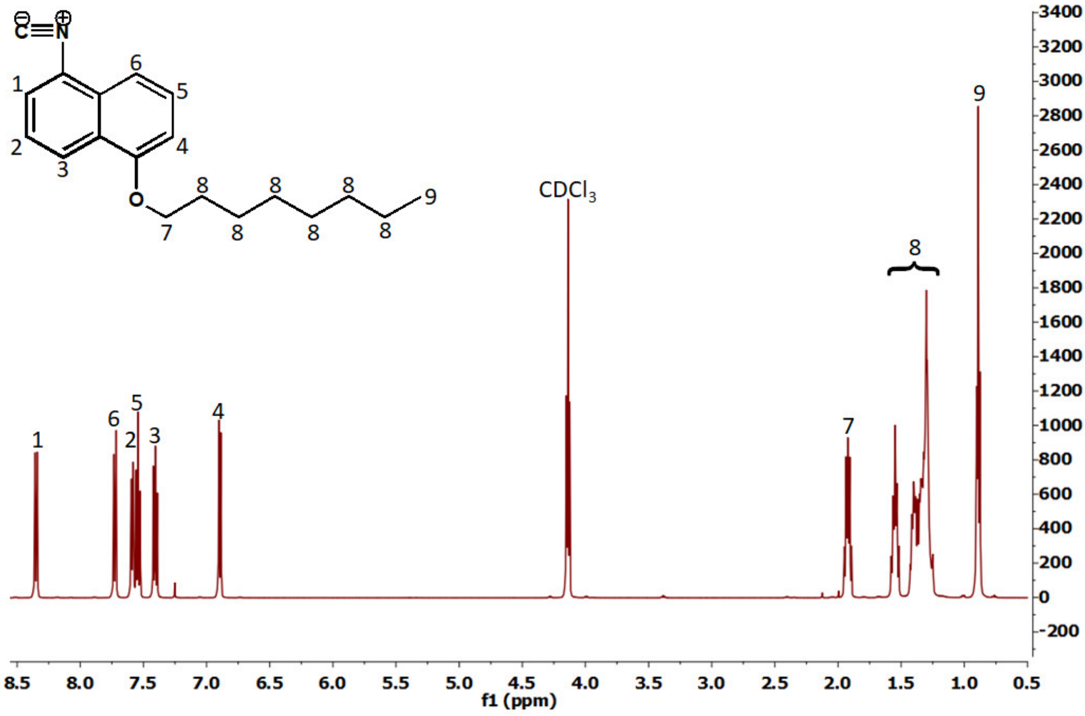

Figure S4. ^{1}H-NMR spectrum of the 5-isocyano-1-(octyloxy)naphthalene (ICON). Chemical shifts (δ_H), ($CDCl_3$, relative to TMS, 500 MHz): 0.89 pm (3H), 1.25-1.55 pm (12H) 1.93 pm (2H), 6.90 pm (1H), 7.41 pm(1H), 7.54 pm (1H), 7.59 pm (1H), 7.73 pm (1H) 8.35 (1H) ppm.

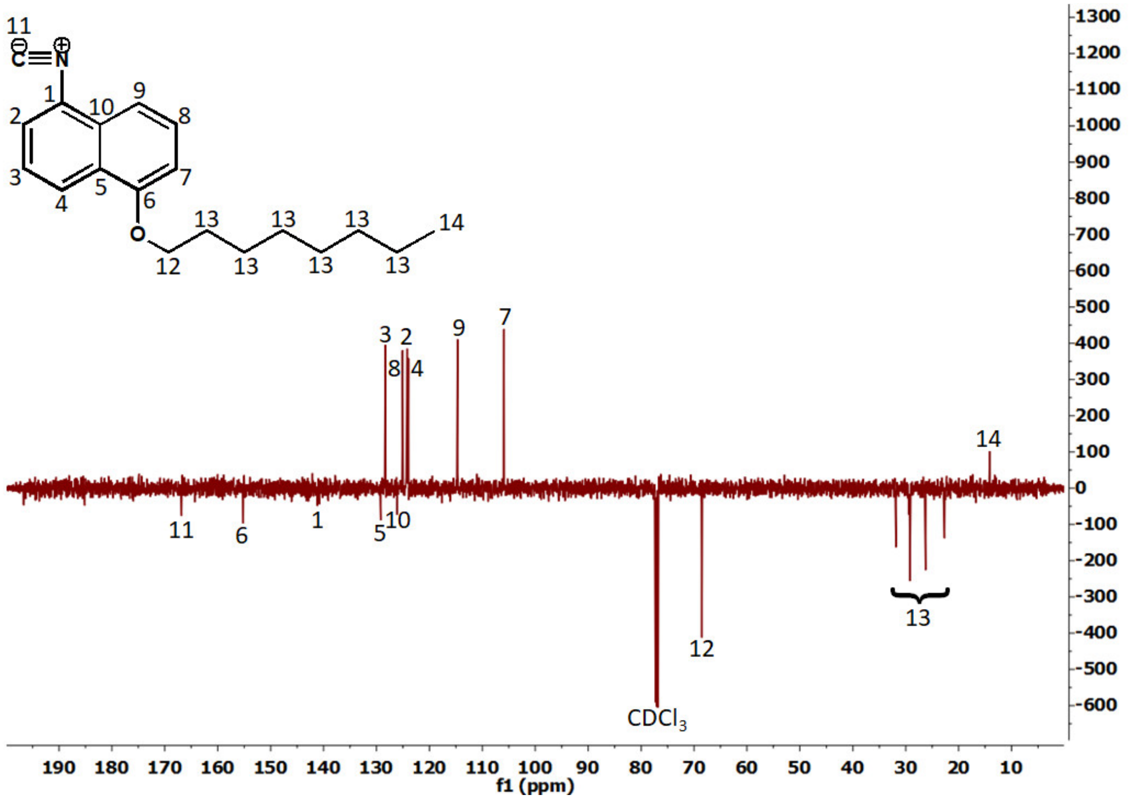

Figure S5. ^{13}C-NMR spectrum of the of 5-isocyano-1-(octyloxy)naphthalene (ICON). Chemical shifts (δ_C), $CDCl_3$, 500 MHz: 14.1 ppm (C14), 22.6 - 31.8 ppm (C13), 68.5 ppm (C12), 105.9 ppm (C7), 114.6 ppm (C9), 123.9 ppm (C4), 124.1 ppm (C2), 125.1 ppm (C8),126.0 ppm (C10), 128.3 ppm (C3), 129.1 ppm (C5), 141.0 ppm (C1), 155.2 ppm (C6), 166.7 ppm (CNC).

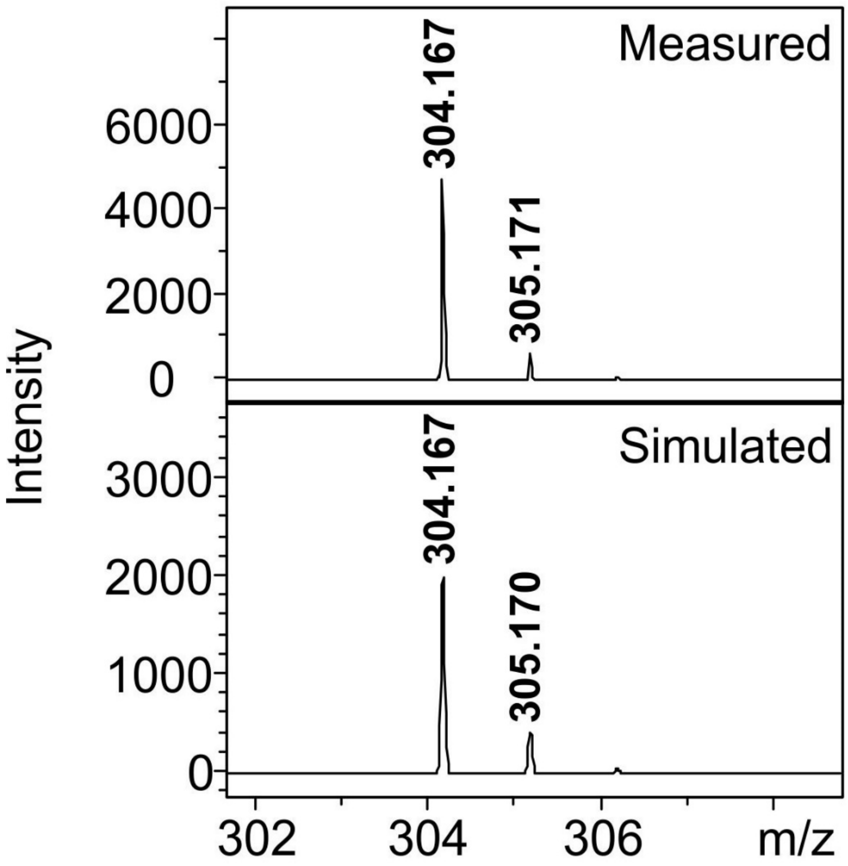

Figure S6. ESI-TOF MS spectrum of the 5-isocyano-1-(octyloxy)naphthalene (ICON).

2. Evaluation of the solvatochromic properties of ICOL and ICON

For the evaluation of the solvatochromic properties of ICOL and ICON derivatives and the determination of their ground-state and excited-state dipole moments various solvatochromic methods including the Weller, Lipper-Mataga, Bilot-Kawski, McRea and Reichardt plots were used.

2.1. The Weller method

The values of the wavenumbers at the maxium emission (ν_f) are plotted as a function of the solvent polarity function as depicted in eq. S1.

$$\nu_f = m_W \Delta f_W(\varepsilon, n) + \text{constant} \qquad \text{(S1)}$$

where m_W is the slope of the line determined by eq. S1, and Δf_W is the solvent polarity function including the refractive index (n) and the permittivity of the solvent (ε) as given by eq. S2.

$$\Delta f_W(\varepsilon, n) = \frac{\varepsilon - 1}{2\varepsilon + 1} - \frac{n^2 - 1}{4n^2 + 2} \qquad \text{(S2)}$$

From the slope of eq. S1 the excited-state dipole moment (μ_E) can be determined by eq. S3

$$\mu_E = \left(2\pi\varepsilon_o m_w hca^3\right)^{1/2} \qquad \text{(S3)}$$

where ε_o is permittivity of the vacuum(8.85×10^{-12} F/m), h is the Planck`s constant (6.62×10^{-34} J/s), c being the speed of the light in vacuum (3×10^{8} m/s), and a stands for the Onsager radius, which was taken to be 0.4 nm (the same value as that was proposed for the 5-isocyano-1-aminonaphthalene).

2.2. The Lippert-Mataga method

The differences in the wavenumbers at the maximum absorption (ν_a) and at the maxium emission (ν_f) are plotted as a function of the solvent polarity function as depicted in eq. S4.

$$\nu_a - \nu_f = m_{LM} \Delta f_{LM}(\varepsilon, n) + \text{constant} \qquad \text{(S4)}$$

where m_{LM} is the slope of the line determined by eq. S4, and Δf_{LM} is the solvent polarity function including the refractive index (n) and the permittivity of the solvent (ε) as given by eq. S5.

$$\Delta f_{LM}(\varepsilon, n) = \frac{\varepsilon - 1}{2\varepsilon + 1} - \frac{n^2 - 1}{2n^2 + 1} \qquad \text{(S5)}$$

From the slope of eq. S4 the difference in the dipole moment of the excited-state and the ground state ($\Delta\mu$) can be determined by eq. S6

$$\mu_E - \mu_G = \Delta\mu = \sqrt{2\pi\varepsilon_o m_{LM} hca^3} \qquad \text{(S6)}$$

where ε_o , h, c and a are the same as indicated in the 2.1.

2.3. The McRea method

The differences in the wavenumbers at the maximum absorption (ν_a) and at the maxium emission (ν_f) are plotted as a function of the solvent polarity function as depicted in eq. S7.

$$\nu_a - \nu_f = m_{MR} \Delta f_{MR}(\varepsilon, n) + \text{constant} \quad \text{(S7)}$$

where m_{MR} is the slope of the line determined by eq. S7, and Δf_{MR} is the solvent polarity function including the refractive index (n) and the permittivity of the solvent (ε) as given by eq. S8.

$$\Delta f_{MR}(\varepsilon, n) = \frac{\varepsilon - 1}{\varepsilon + 2} - \frac{n^2 - 1}{n^2 + 2} \quad \text{(S8)}$$

From the slope of eq. S8 the difference in the dipole moment of the excited-state and the ground state ($\Delta\mu$) can be determined by eq. S9

$$\mu_E - \mu_G = \Delta\mu = \sqrt{2\pi\varepsilon_o m_{MR} hca^3} \quad \text{(S9)}$$

where ε_o , h, c and a are the same as indicated in the 1.1.

2.4. The Bilot-Kawski method

The differences in the wavenumbers at the maximum absorption (ν_a) and at the maximum emission (ν_f), as well as ν_a+ν_f are plotted as a function of the solvent polarity functions Δf_{BK_1} and Δf_{BK_2} as depicted in eq. S10 and eq. S11.

$$\nu_a - \nu_f = m_{BK\_1} \Delta f_{BK\_1}(\varepsilon, n) + \text{constant} \quad \text{(S10)}$$

$$\nu_a + \nu_f = m_{BK\_2} \Delta f_{BK\_2}(\varepsilon, n) + \text{constant} \quad \text{(S11)}$$

where m_{BK_1} and m_{BK_2} are the slopes of the line determined by eq. S10 and eq. S11, respectively, and Δf_{BK_1} and Δf_{BK_2} are the solvent polarity functions including the refractive index (n) and the permittivity of the solvent (ε) as given by eq. S12 and eq. S13, respectively.

$$\Delta f_{BK\_1}(\varepsilon, n) = \frac{2n^2 + 1}{n^2 + 2}\left(\frac{\varepsilon - 1}{\varepsilon + 2} - \frac{n^2 - 1}{n^2 + 2}\right) \quad \text{(S12)}$$

$$\Delta f_{BK\_2}(\varepsilon, n) = \frac{2n^2 + 1}{2n^2 + 4}\left(\frac{\varepsilon - 1}{\varepsilon + 2} - \frac{n^2 - 1}{n^2 + 2}\right) + 1.5\left[\frac{n^2 - 1}{\left(n^2 + 2\right)^2}\right] \quad \text{(S13)}$$

From the slopes of eq. S12 and eq. S13 the excited-state and the ground-state dipole moments can be determined by eq. S14 and eq. S15, respectively.

$$\mu_E = \frac{m_{BK\_1} - m_{BK\_2}}{2}\sqrt{\frac{2\pi\varepsilon_o hca^3}{m_{BK\_1}}} \tag{S14}$$

$$\mu_G = \frac{m_{BK\_1} + m_{BK\_2}}{2}\sqrt{\frac{2\pi\varepsilon_o hca^3}{m_{BK\_1}}} \tag{S15}$$

2.5. The Reichardt method

The differences in the wavenumbers at the maximum absorption (ν_a) and at the maximum emission (ν_f) are plotted as a function of the empirical polarity values (E_T^N) as depicted in eq. S16.

$$\nu_a - \nu_f = 11307.6\left(\frac{\Delta\mu^2 a_B^3}{\Delta\mu_B{}^2 a^3}\right)E_T^N + \text{constant} \tag{S16}$$

where $\Delta\mu_B$ and a_B are the dipole moment change (9 D) and the Onsager radius of the reference betain molecule (0.62 nm), respectively.

From the slopes of eq. S16 and eq. S13 the difference in the dipole moment of the excited-state and the ground state ($\Delta\mu$) can be deduced from eq. S17.

$$\mu_E - \mu_G = \Delta\mu = \sqrt{\frac{81 \times m_{E_T^N}}{11307.6\left(\frac{a_B}{a}\right)^3}} \tag{S17}$$

2.6. The refractive index and the permittivity values and the Kamlet-Taft parameters used for the evaluation (and Table S1 and Table S2)

Table S1. The refractive index (n) and the permittivity (ε) of the solvents used in this study.

No	Solvent	n	ε
**1**	1,4-dioxane	1.422	2.25
**2**	2-propanol	1.378	17.9
**3**	Acetonitrile	1.344	37.5
**4**	Chloroform	1.448	4.81
**5**	Dichloromethane	1.425	8.93
**6**	Dimethylsulfoxide	1.480	46.7
**7**	Ethyl acetate	1.372	6.02
**8**	Methanol	0.98	0.66
**9**	N,N-Dimethylformamide	1.430	36.71
**10**	Tetrahydrofuran	1.407	7.58
**11**	Toluene	1.497	2.38

Table S2. The Kamlet-Taft (α, β, π*) parameters and E^N_T values for the solvents used in this study.

No	Solvent	α	β	π*	E^N_T
**1**	1,4-dioxane	0.00	0.37	0.49	0.164
**2**	2-propanol	0.76	0.84	0.48	0.546
**3**	Acetonitrile	0.19	0.40	0.66	0.46
**4**	Chloroform	0.20	0.10	0.69	0.259
**5**	Dichloromethane	0.13	0.10	0.73	0.309
**6**	Dimethylsulfoxide	0.00	0.76	1.00	0.444
**7**	Ethyl acetate	0.00	0.45	0.45	0.228
**8**	Methanol	0.98	0.66	0.60	0.762
**9**	N,N-Dimethylformamide	0.00	0.69	0.88	0.386
**10**	Tetrahydrofuran	0.00	0.55	0.55	0.207
**11**	Toluene	0.00	0.11	0.49	0.099

Data form Reichardt C, Welton T, *Solvents and Solvent Effects in Organic Chemistry, 4th ed.*, Wiley-VCH, Weinheim, 2010; pp. 471 (Table 7.4.).

3. The results of additional TD-DFT calculations (and Table S3-S7 and Fig. S7-S13)

Table S3. The cavity volumes obtained for the optimized geometries using PCM solvation with its Gaussian16 default parameters. *The alkyloxy group of the ICON molecule was truncated to a methoxy group in the calculations.

No	molecule	state	solvent	cavity volume (Å^3)
1	ICOL	ground	toluene	237.506
2	ICOL	S_1	toluene	238.069
3	ICOL-DMSO	ground	DMSO	346.609
4	ICOL	S_1	DMSO	238.130
5	ICON*	ground	toluene	262.402
6	ICON*	S_1	toluene	263.412
7	ICON*	ground	DMSO	262.463
8	ICON*	S_1	DMSO	263.461
9	$ICOL^-$	S_1	toluene	234.752
10	$ICOL^-$	S_1	DMSO	234.638

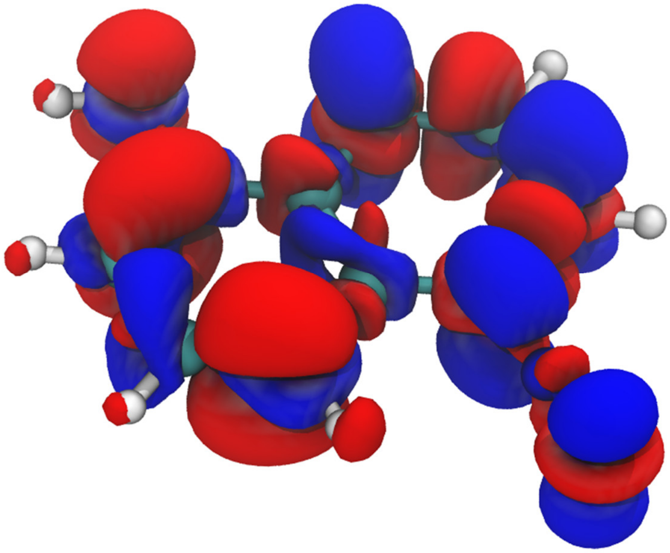

Figure S7. The electron density difference between the vertical excited-state and the ground-state of ICOL in toluene. The red and blue surfaces correspond to isovalues -0.0015 and 0.0015 electrons/bohr^3, respectively.

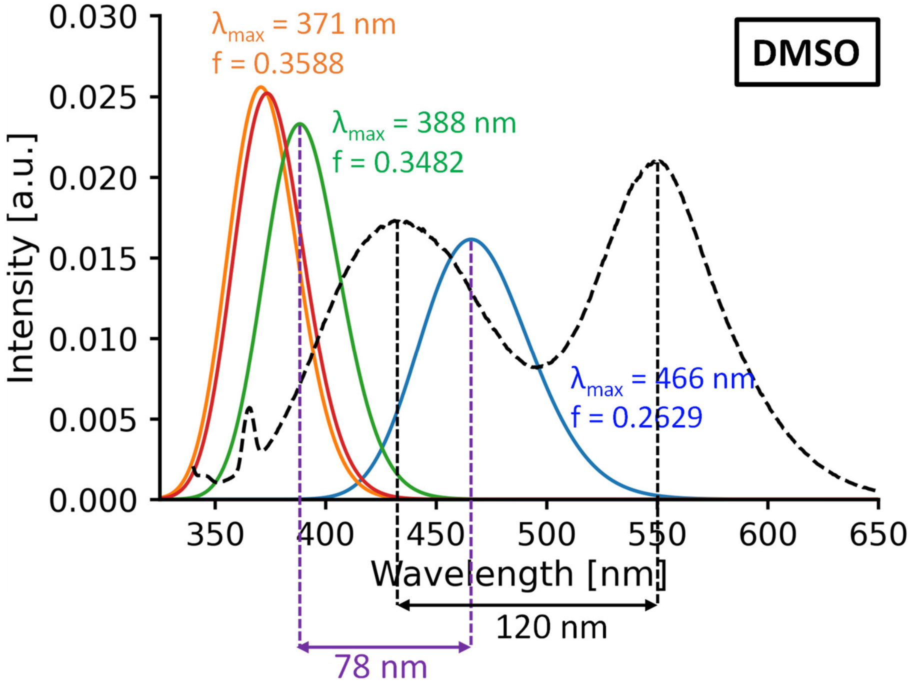

Figure S8. The spectra shown in Fig. 2 where the calculations were performed with the CAM-B3LYP functional. Note that there are significant errors both in the peak positions and in reproducing the shift associated with deprotonation.

Table S4. Comparison of the M06 and CAM-B3LYP functionals in reproducing the experimental absorption wavelength.

	**M06**		**CAM-B3LYP**		**expt.**
	**λ (nm)**	**oscillator strength**	**λ (nm)**	**oscillator strength**	**λ (nm)**
**ICOL (toluene)**	329	0.1784	295	0.2299	320
**ICOL (DMSO)**	334	0.1690	–	–	329
**ICOL-DMSO (DMSO)**	346	0.1674	309	0.2282	329

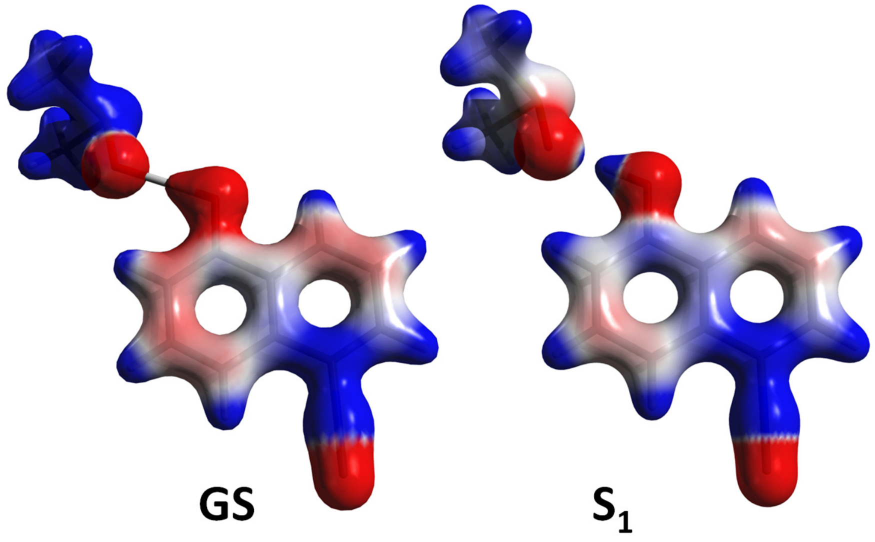

Figure S9. The electrostatic potentials mapped on the electron densities of the ground-state (GS) and the relaxed first singlet excited-state (S1) of ICOL in DMSO. Red areas correspond to negatively charged regions while blue areas indicate positive charges. Isovalue = 0.1 electrons/bohr^3.

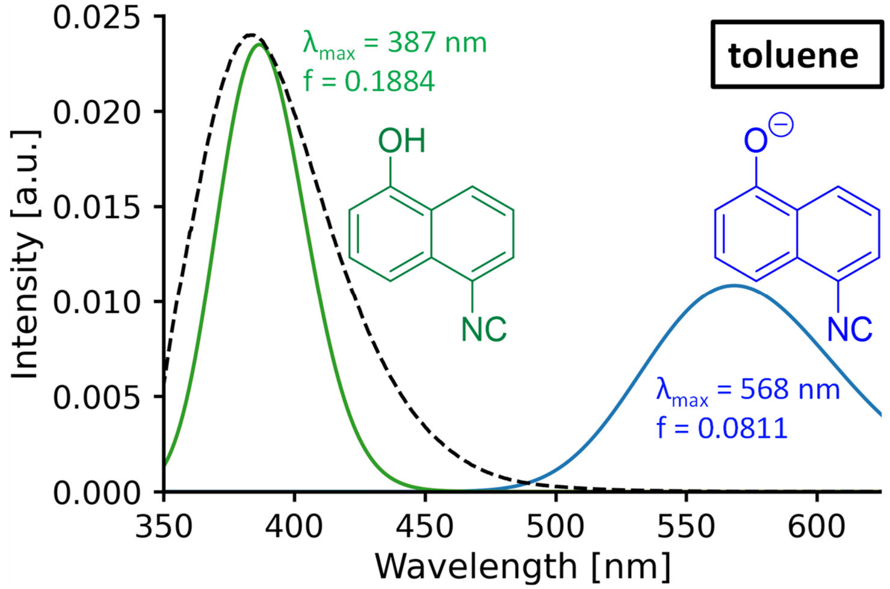

Figure S10. The calculated emission spectra of ICOL in toluene, showing only the first emission peaks for all indicated species. All the spectra were normalized to unit area. The green and blue lines correspond to the neutral and deprotonated forms of ICOL using only implicit solvation. The dashed black line corresponds to the experimental spectrum, which is rescaled to match the maximum height of the calculated spectra.

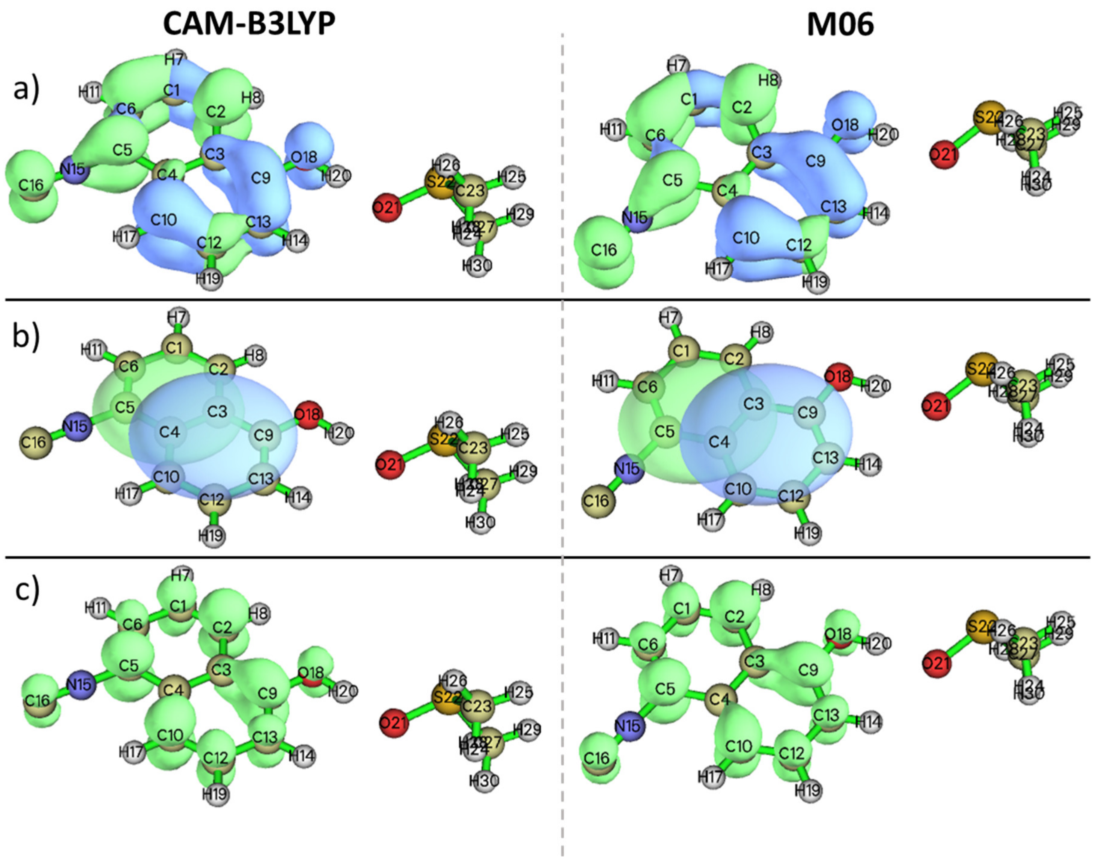

Figure S11. Isosurfaces calculated with the CAM-B3LYP and M06 functionals for a) the hole (blue) and electron (green) distributions, b) the C_{hole} and C_{ele} functions, c) the hole and electron overlap (S_r) function of the ICOL-DMSO adduct in DMSO. The isovalue of 0.002 electrons/bohr^3 was used throughout.

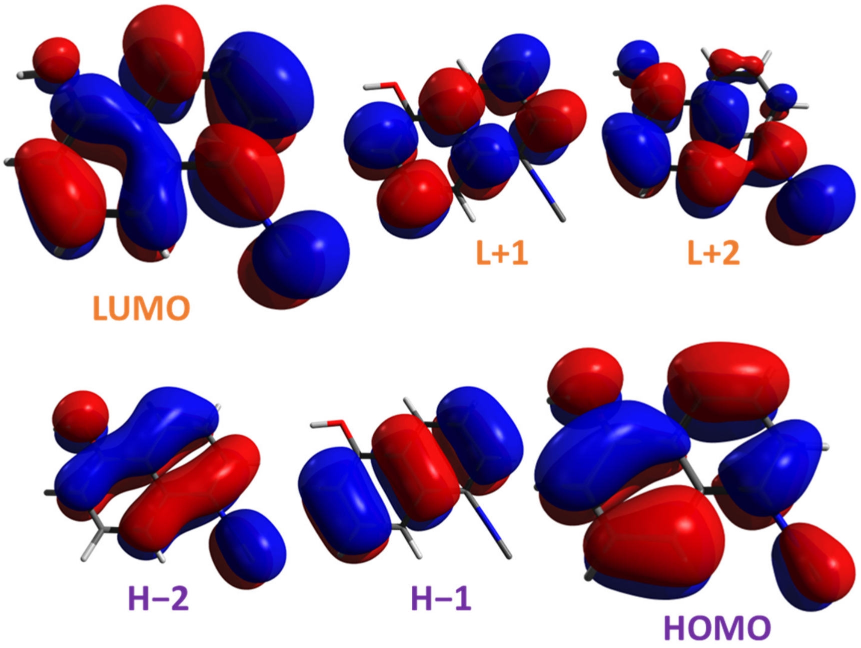

Figure S12. Frontier orbitals of ICOL in toluene.

Table S5. Parameters calculated for the first three excitations of ICOL in toluene.

excited-state No.	λ (nm)	oscillator strength	Δr (Å)	Λ	orbitals involved	
**1**	329	0.1784	1.1784	0.7933	H → L	97.1%
**2**	290	0.0237	0.6259	0.6232	H → L+1	63.7%
**3**	239	0.2175	0.5783	0.7636	H−2 → L<br>H−1 → L<br>H → L+1<br>H → L+2	70.1%<br>12.4%<br>7.0%<br>5.5%

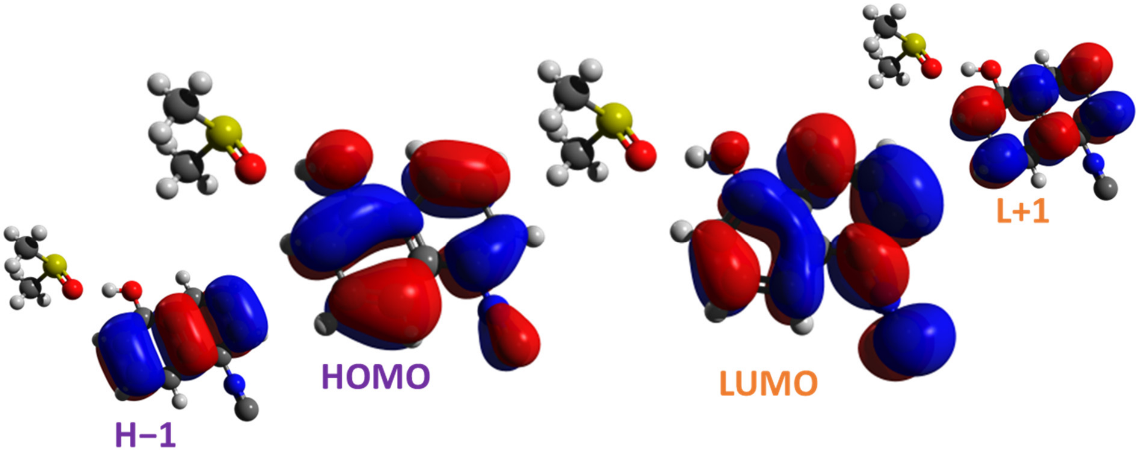

Figure S13. Frontier orbitals of the ICOL-DMSO adduct in DMSO, calculated with the CAM-B3LYP functional.

Table S6. Parameters calculated for the first three excitations of the ICOL-DMSO adduct in DMSO, calculated with the CAM-B3LYP functional.

excited-state No.	λ (nm)	oscillator strength	Δr (Å)	Λ	orbitals involved	
**1**	309	0.2282	1.4792	0.7484	H → L	97.1%
**2**	277	0.0380	0.8221	0.6159	H → L+1<br>H−1 → L	67.0%<br>29.9%
**3**	225	0.8871	0.9636	0.5926	H−1 → L<br>H → L+1<br>H−3 → L+1	60.8%<br>28.2%<br>5.6%

Table S7. M06/TZVP + PCM (toluene, DMSO) optimized geometries in XYZ format

```
20
ICOL ground state in toluene
C                    0.77372900    2.41151200   -0.00021300
C                   -0.50177200    1.91878800   -0.00025100
C                   -0.73653300    0.52947800   -0.00046200
C                    0.35601900   -0.37797200    0.00030500
C                    1.66083900    0.18269200    0.00001800
C                    1.86774400    1.53788800   -0.00003700
H                    0.94674500    3.48049800   -0.00017600
H                   -1.35107500    2.59000200   -0.00046100
C                   -2.05265600   -0.00380100    0.00018200
C                    0.11980900   -1.76752100   -0.00019200
H                    2.88224700    1.91682500   -0.00009800
C                   -1.16315700   -2.23614000   -0.00012400
C                   -2.25713500   -1.35794600   -0.00048600
H                   -3.26991000   -1.74893400    0.00015500
N                    2.75008600   -0.66713000    0.00019400
C                    3.65781400   -1.40313700    0.00025300
```

```
H                  0.95787900   -2.45306800    0.00032100
O                 -3.06361000    0.89412500    0.00064700
H                 -3.90801300    0.43248400    0.00039600
H                 -1.34780800   -3.30394400   -0.00062900

20
ICOL S1 in toluene
C                  0.87768600    2.40459200   -0.00798400
C                 -0.46060800    1.95350900   -0.00878400
C                 -0.71868000    0.57337400   -0.01119000
C                  0.34422200   -0.36606200    0.00497100
C                  1.68124500    0.12600800    0.00173900
C                  1.92834300    1.53059800   -0.00156800
H                  1.07253700    3.47084600   -0.01020200
H                 -1.27947800    2.65860800   -0.01531200
C                 -2.03714300    0.04855900    0.00610900
C                  0.05556500   -1.73848800   -0.01244800
H                  2.95382300    1.87702300   -0.00110000
C                 -1.26580800   -2.22629500   -0.00343500
C                 -2.30607800   -1.34119500   -0.01090500
H                 -3.33773800   -1.67773700   -0.00281700
N                  2.71879300   -0.74510900    0.00820200
C                  3.61450500   -1.51255100    0.01253800
H                  0.87840500   -2.44380900   -0.01562100
O                 -3.03301100    0.92884400    0.02039900
H                 -3.88808800    0.48072500    0.02363000
H                 -1.44642100   -3.29293500   -0.01345100

23
ICOL-OMe ground state in toluene
C                  1.03825900    2.42586900   -0.00001000
C                 -0.21231100    1.87082800   -0.00001100
C                 -0.38065200    0.47265400   -0.00000400
C                  0.75512700   -0.37743300    0.00000700
C                  2.03132200    0.24444500    0.00000800
C                  2.17351600    1.60837400   -0.00000000
H                  1.15796200    3.50222100   -0.00001600
H                 -1.09255500    2.50029800   -0.00001900
C                 -1.67643800   -0.12290800   -0.00000500
C                  0.58753600   -1.77804500    0.00001300
H                  3.16840100    2.03605500    0.00000100
C                 -0.66973800   -2.30627200    0.00001100
C                 -1.81243800   -1.48758400    0.00000100
H                 -2.79119600   -1.94831900    0.00000100
N                  3.15997200   -0.55255900    0.00001700
C                  4.10168000   -1.24455800    0.00002500
H                  1.45809900   -2.42184300    0.00002100
O                 -2.70423800    0.74907600   -0.00001200
H                 -0.80184000   -3.38202300    0.00001500
C                 -4.01635100    0.23430100   -0.00002500
H                 -4.20852600   -0.37081200    0.89215800
H                 -4.68481000    1.09251400   -0.00003600
H                 -4.20850500   -0.37082100   -0.89220600

23
ICOL-OMe S1 in toluene
C                  1.10879100    2.42800900   -0.00012900
C                 -0.19899500    1.89407500   -0.00018500
C                 -0.37229900    0.50231000   -0.00018800
C                  0.74774000   -0.36646900    0.00008500
C                  2.05480000    0.20795600    0.00008300
C                  2.21301000    1.62222100    0.00001800
H                  1.23619100    3.50449500   -0.00017400
H                 -1.05908300    2.54756700   -0.00031300
C                 -1.66587500   -0.10222300   -0.00003200
C                  0.54673500   -1.75313200   -0.00012600
H                  3.21457600    2.03295700    0.00006100
C                 -0.74107600   -2.32058700   -0.00006700
```

```
C                 -1.84123200   -1.50768500   -0.00020100
H                 -2.83611000   -1.93124600   -0.00016900
N                  3.14266100   -0.59926000    0.00022600
C                  4.07999900   -1.31567900    0.00033700
H                  1.41181600   -2.40611000   -0.00011000
O                 -2.68324800    0.74549200    0.00006900
H                 -0.85438100   -3.39666600   -0.00017500
C                 -4.02162300    0.27201000    0.00012500
H                 -4.22681900   -0.32107900    0.89463900
H                 -4.65177500    1.15714700    0.00019500
H                 -4.22692200   -0.32100800   -0.89441300

19
deprotonated ICOL S1 in toluene
C                  0.85250800    2.40424200   -0.00178800
C                 -0.47208000    1.98846000   -0.00256800
C                 -0.74821000    0.58673600   -0.00274500
C                  0.28830100   -0.34075100    0.00092700
C                  1.64533100    0.11222700    0.00083900
C                  1.90664000    1.50955200   -0.00033200
H                  1.07549100    3.46785200   -0.00219500
H                 -1.29497200    2.68931700   -0.00282300
C                 -2.13121400    0.12137000    0.00093200
C                 -0.00535000   -1.74304500   -0.00270600
H                  2.93490200    1.85069900    0.00009900
C                 -1.30770700   -2.20461000   -0.00046700
C                 -2.35315900   -1.30468000   -0.00359100
H                 -3.38762000   -1.63038300   -0.00185700
N                  2.66350200   -0.78019000    0.00216800
C                  3.56980300   -1.54356600    0.00310400
H                  0.82068700   -2.44364900   -0.00199600
O                 -3.09507000    0.91044500    0.00589900
H                 -1.50162100   -3.27167000   -0.00323200

30
ICOL-DMSO adduct ground state in DMSO
C                -3.10914300    2.37666100    0.15185300
C                -1.81292100    1.94635700    0.06495000
C                -1.50885000    0.57419800   -0.03108500
C                -2.55637300   -0.38600600   -0.03768800
C                -3.88452200    0.10990200    0.05453100
C                -4.15881700    1.45048600    0.14688900
H                -3.33244500    3.43371300    0.22512600
H                -0.99702800    2.65798100    0.06841100
C                -0.16116000    0.11914300   -0.12039700
C                -2.25396600   -1.75845700   -0.13425400
H                -5.18898900    1.77743500    0.21506200
C                -0.94704600   -2.15505600   -0.21910400
C                 0.10371100   -1.22763200   -0.21504400
H                 1.13425400   -1.55881400   -0.28299400
N                -4.93220200   -0.79278700    0.05108100
C                -5.80151000   -1.57180300    0.04646300
H                -3.05439700   -2.48782600   -0.13936500
O                 0.79475600    1.05463300   -0.11053300
H                -0.71102500   -3.21068000   -0.29369500
H                 1.69351000    0.63712000   -0.16756300
O                 3.15661200   -0.15220000   -0.29373500
S                 4.48432300    0.43371900    0.22723700
C                 5.15054400   -0.85390500    1.31117300
H                 5.13467400   -1.79981400    0.76926000
H                 6.16462400   -0.58399100    1.60433700
H                 4.50746800   -0.90256500    2.18752000
C                 5.64591100    0.20016300   -1.14089300
H                 5.34612900    0.87621600   -1.93893900
H                 6.65162100    0.44450300   -0.80017100
H                 5.58175800   -0.83701900   -1.47058300
```

```
30
ICOL-DMSO adduct S1 in DMSO
C                 -3.12604200    2.38000800    0.17712300
C                 -1.77918800    1.96855600    0.07707100
C                 -1.48632100    0.59964400   -0.03643000
C                 -2.52076200   -0.36775300   -0.03838000
C                 -3.87244100    0.08389400    0.06151400
C                 -4.15348900    1.47768100    0.17181800
H                 -3.34903500    3.43772700    0.26172400
H                 -0.97837200    2.69423200    0.08160300
C                 -0.14233800    0.12483600   -0.12668700
C                 -2.19724600   -1.72750400   -0.16630900
H                 -5.18547300    1.79664300    0.24863000
C                 -0.86635300   -2.17127800   -0.26023400
C                  0.15168700   -1.26049000   -0.25586200
H                  1.19162800   -1.55814900   -0.32607700
N                 -4.88479900   -0.81359400    0.05599600
C                 -5.75907600   -1.60691800    0.04869500
H                 -2.99823600   -2.45738700   -0.17854400
O                  0.81502000    1.01495400   -0.10269600
H                 -0.65826300   -3.22881900   -0.35549900
H                  1.73600800    0.59518300   -0.17239000
O                  3.10315800   -0.13557600   -0.32873700
S                  4.40802500    0.40787100    0.30181100
C                  5.08935500   -1.01442300    1.18581200
H                  5.11315800   -1.86256500    0.50146200
H                  6.08844100   -0.76580800    1.54216000
H                  4.43124900   -1.21700900    2.02812700
C                  5.58356600    0.41767400   -1.07147200
H                  5.26205000    1.19073100   -1.76617600
H                  6.57666500    0.64871600   -0.68735100
H                  5.56182900   -0.56285000   -1.54711300
```

4. Derivation of the expression for the determination of pK_a and the apparent pK_a*values (and Fig. S14 and Fig. S15)

Approach I. (using absorbance values at one wavelength, λ)

For an acid HA equilibrium S17 can be written and the corresponding K_a is given by eq. S18.

$$HA \rightleftharpoons H^+ + A^- \ (K_a) \quad \text{(S17)}$$

$$K_a = \frac{[H^+][A^-]}{[HA]} \quad \text{(S18)}$$

The absorbance due to the neutral and the anionic forms at a given wavelength (λ) comes as:

$$Abs(\lambda) = \varepsilon_{HA}[HA] + \varepsilon_A[A^-] \quad \text{(S19)}$$

where ε_{HA} and ε_A are the molar absorbance coefficients at λ for the neutral and the anionic form, respectively.

Taking into account that $c_o = [HA] + [A^-]$ and $\frac{K_a}{[H^+]} = \frac{[A^-]}{[HA]}$ from eq. S18, eq. S19 read now as eq. S20.

$$\mathrm{Abs}(\lambda) = \frac{\varepsilon_{HA} c_o}{1 + \frac{K_a}{[H^+]}} + \frac{\varepsilon_A [A^-] \frac{K_a}{[H^+]}}{1 + \frac{K_a}{[H^+]}} \tag{S20}$$

Furthermore, using $\varepsilon_{HA} c_o = p_1$ and $\varepsilon_A c_o = p_2$ and $\frac{K_a}{[H^+]} = 10^{pH-pK_a}$ and substituting them into eq. S20, eq. S21 comes as:

$$\mathrm{Abs}(\lambda) = \frac{p_1}{1 + 10^{pH-pK_a}} + \frac{p_2 10^{pH-pK_a}}{1 + 10^{pH-pK_a}} \tag{S21}$$

Eq. S21 can be fitted to the experimental Abs(λ) *versus* pH data and the value of pK_a can be determined.

Approach II. (using the ratios of absorbances at two wavelengths, ratiometric)

For wavelength 1 (λ_1) eq. S22 can be written using eq. S3.

$$\mathrm{Abs}(\lambda_1) = \varepsilon_{HA,1}[HA] + \varepsilon_{A,1}[A^-] \tag{S22}$$

Similarly, for wavelength 2 (λ_2) eq. S23 comes:

$$\mathrm{Abs}(\lambda_2) = \varepsilon_{HA,2}[HA] + \varepsilon_{A,2}[A^-] \tag{S23}$$

where $\varepsilon_{HA,1}$ and $\varepsilon_{HA,2}$, $\varepsilon_{A,1}$ and $\varepsilon_{A,2}$ are the molar extinction coefficients (or molar emissivity in the case of fluorescence) at λ_1 and λ_2 for the neutral and the anionic form, respectively.

Thus, composing the ratio of Abs(l1) to Abs(l1) + Abs(l2) one can get eq. S24:

$$\frac{\mathrm{Abs}(\lambda_1)}{\mathrm{Abs}(\lambda_1) + \mathrm{Abs}(\lambda_2)} = \frac{\varepsilon_{HA,1} + \varepsilon_{A,1}\frac{K_a}{[H^+]}}{\varepsilon_{HA,1} + \varepsilon_{A,1}\frac{K_a}{[H^+]} + \varepsilon_{HA,2} + \varepsilon_{A,2}\frac{K_a}{[H^+]}} \tag{S24}$$

Rearranging eq. S24, eq S25 can be obtained:

$$\frac{\mathrm{Abs}(\lambda_1)}{\mathrm{Abs}(\lambda_1) + \mathrm{Abs}(\lambda_2)} = \frac{1 + p_1 10^{pH-pK_a}}{1 + 10^{pH-pK_a}(p_1 + p_2) + p_3} \tag{S25}$$

where $p_1 = \varepsilon_{A,1}/\varepsilon_{HA,1}$; $p_2 = \varepsilon_{A,2}/\varepsilon_{HA,1}$ and $p_3 = \varepsilon_{HA,2}/\varepsilon_{HA,1}$;

Fitting eq. S25 to the experimental curves, pK_a value can be determined. Since in the case of fluorescence spectra it was observed that on increasing the acidity of the solution the emission intensities decreased, most probably due to the quenching of by H^+ ions. Therefore, only approach II. gave acceptable results in this case. However, in order to use the same approach approach II was also employed for the determination of the ground-state pK_a although approach I. yielded very similar results.

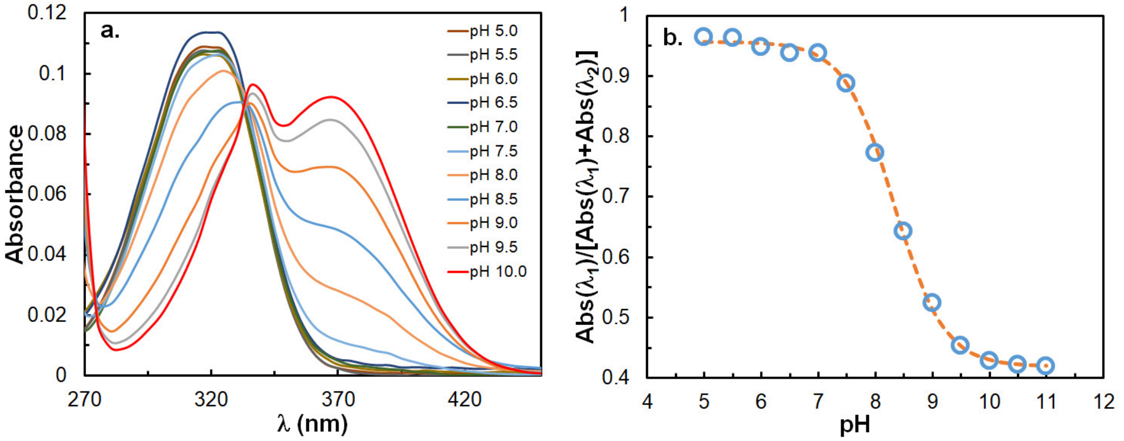

Figure S14. Variation of the electronic absorption spectrum (a) and the value of the $Abs(\lambda_1)/(Abs(\lambda_1)+Abs(\lambda_2)$ of ICOL as a function of pH. The concentration of ICOL is 19.7 μM and λ_1= 325 nm and λ_2= 365 nm.

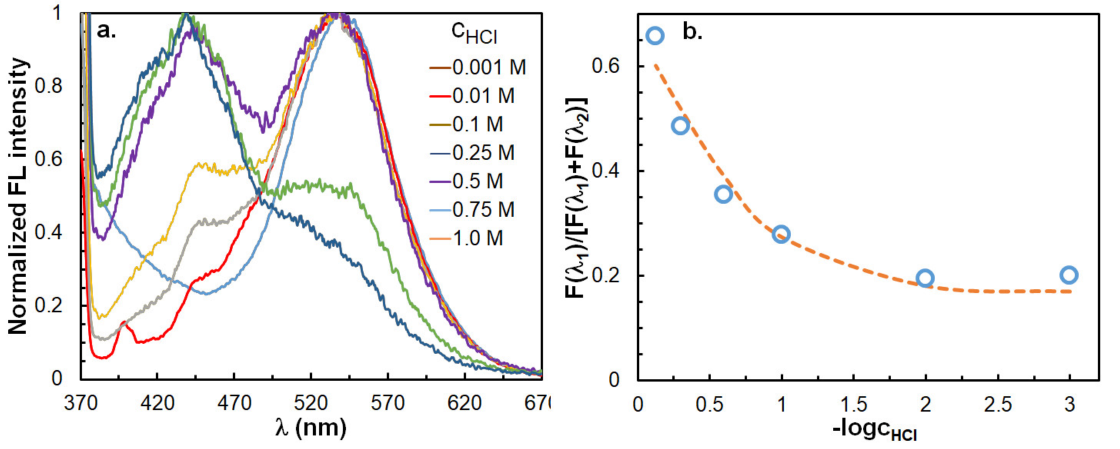

Figure S15. Variation of the normalized emission spectrum (a) and the value of the $F(\lambda_1)/(F(\lambda_1)+F(\lambda_2)$ (b) of ICOL as a function of HCl concentration. The concentration of ICOL is 50.6 μM. and and λ_1= 440 nm and λ_2= 540 nm.

Estimation of the excited-state pK$_a^*$ value by the Förster cycle

The excited-state pK$_a^*$ value can be estimated using the Förstler-equation shown in eq. S26

$$pK_a - pK_a^* = \frac{N_A h}{2.3RT}\left(\nu_{avg,HA} - \nu_{avg,A^-}\right) \tag{S26}$$

where pK$_a$ is the ground-state acidic dissociation constant, N_A and h are the Avagadro`s number and the Planck`s constant, respectively, while $\nu_{avg,HA}$ and ν_{avg,A^-} are the average wavenumber for the absorption and emission of the neutral and the anionic forms, respectively, i.e., $\nu_{avg,HA} = (\nu_{a,HA} + \nu_{f,HA})/2$ and $\nu_{avg,A^-} = (\nu_{a,A^-} + \nu_{f,A^-})/2$.

5. Derivation of eq. (6) and emission intensities *versus* surfactant concentrations (and Fig. S16-S19)

The partition coefficient of the fluorophore molecules into the micelles (P_m) can be given as:

$$P_m = \frac{[FL]_m}{[FL]_w} \tag{S27}$$

where $[FL]_m$ and $[FL]_w$ is the concentration of the fluorophore in the micelles and water, respectively.

whereas, the micelle volume to the total volume of the solution (α_m) is obtained as:

$$\alpha_m = (c - cmc)M_d / \rho_d \tag{S28}$$

where c and cmc are the concentration and the critical micelle concentration (in mol/L), M_d and ρ_d is the molecular weight (in g/mol) and the density (in g/L) of the detergent.

Let the concentration of a fluorophore (FL) in water and in micelles be $[FL]_w$ and $[FL]_m$, respectively, and its initial concentration is $[FL]_o$. According to the mass conservation eq. S29 will be valid:

$$\left([FL]_o - [FL]_w\right)V_w = [FL]_m V_m \tag{S29}$$

where V_w and V_m are the volumes of water and micelle phase, respectively.

Thus, the total emissions coming from the aqueous and the micellar phase can be expressed by eq. S30 as:

$$F = \varepsilon_w [FL]_w V_w + \varepsilon_m [FL]_m V_m \tag{S30}$$

where ε_w and ε_m are the molar emissivities of the fluorophore in aqueous and micellar phase.

When all the fluorophore molecules are incorporated into the micellar phase, then the fluorescence intensity can be given by eq. S31 and substituting it into eq. S30, eq. S32 comes as:

$$F_\infty = \varepsilon_m [FL]_o V_w \tag{S31}$$

$$\frac{F}{F_\infty} = \frac{\varepsilon_w}{\varepsilon_m}\frac{[FL]_w}{[FL]_o} + \frac{[FL]_m}{[FL]_o}\frac{V_m}{V_w} \tag{S32}$$

Furthermore, using $\alpha_m = \frac{V_m}{V_m + V_w}$, $\frac{V_m}{V_w} = \frac{\alpha_m}{1 - \alpha_m}$ and $P_m = \frac{[FL]_m}{[FL]_w}$, and substituting them into eq. S32, we get eq S33 as:

$$\frac{F}{F_\infty} = \frac{\alpha_m}{\alpha_m + \frac{1-\alpha_m}{P_m}} + \frac{\varepsilon_w}{P_m \varepsilon_m}\frac{(1-\alpha_m)}{\alpha_m + \frac{1-\alpha_m}{P_m}} \tag{S33}$$

Rearranging eq. S33 and using $\beta = \frac{\varepsilon_w}{\varepsilon_m}$, eq. S34 can be obtained which is the same as eq. 1.

$$F = \frac{F_\infty}{\alpha_m + \frac{1-\alpha_m}{P_m}}\left[\alpha_m + \frac{\beta}{P_m}(1-\alpha_m)\right] \tag{S34}$$

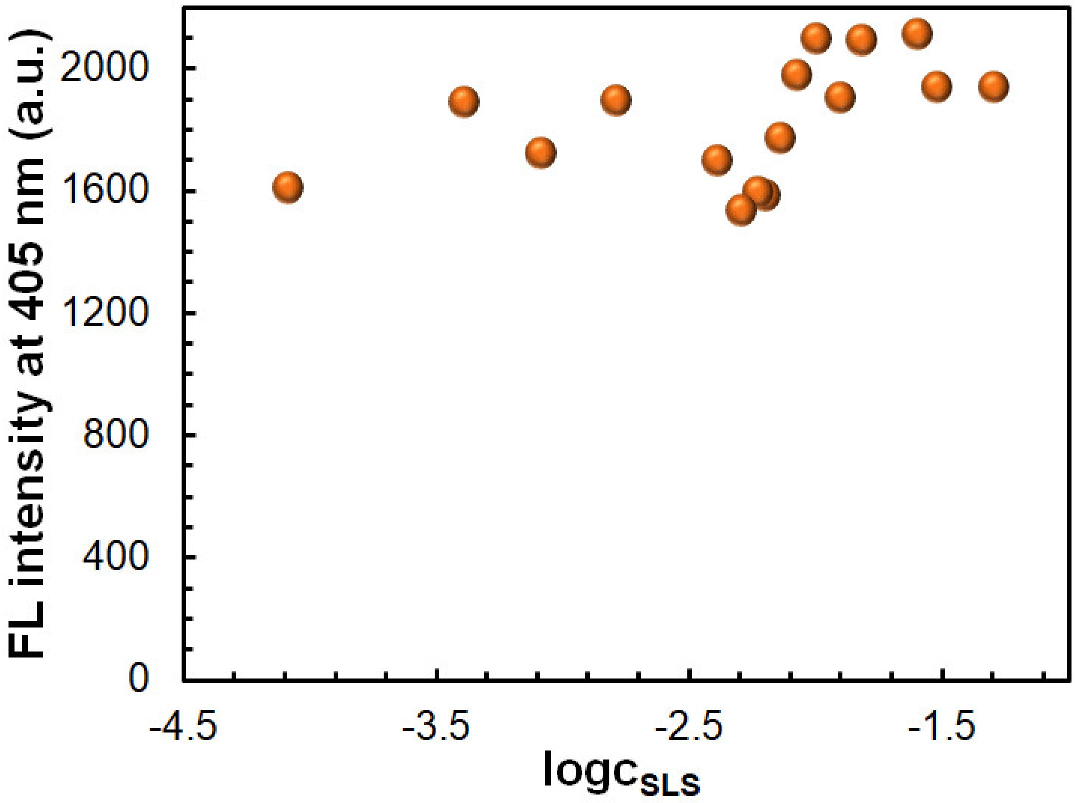

Figure S16. Variation of the fluorescence intensity at 405 nm for the aqueous solution of 5-isocyano-1-(octyloxy)naphthalene (ICON) in the presence of sodium lauryl sulfate (SLS) at different concentrations. The concentration of ICON is 50.6 μM.

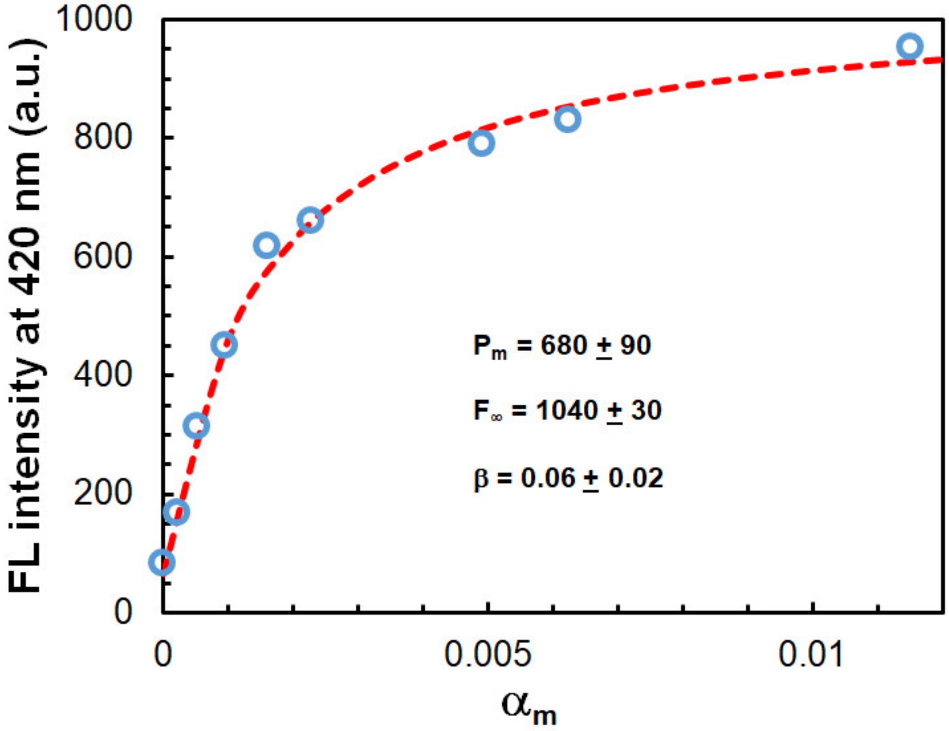

Figure S17. Variation of the fluorescence intensity (FL) at 420 nm with α_m. for the ICOL-SLS micellar system.

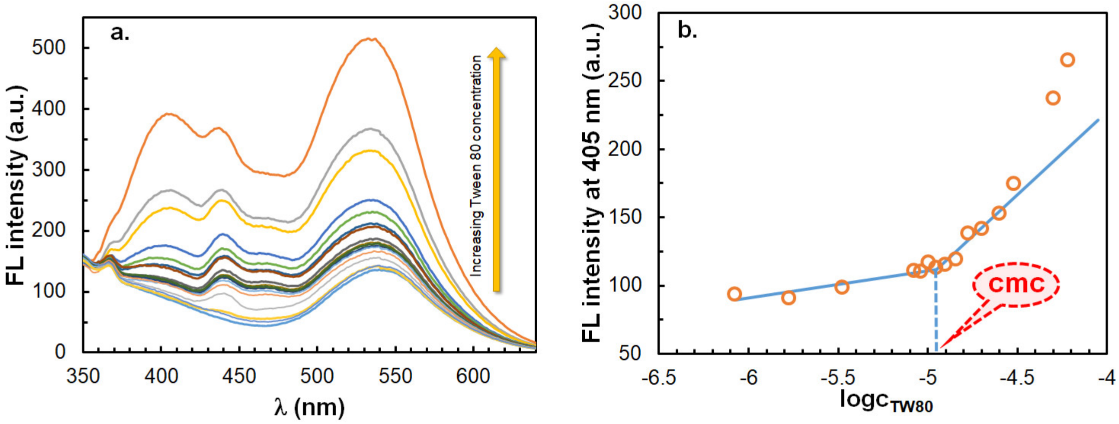

Figure S18. Variation of the emission spectrum (a) and the fluorescence intensity at 405 nm (b) for the aqueous solution of 5-isocyanonaphthalene-1-ol (ICOL) in the presence of Tween 80 (TW80) at different concentrations. The concentration of ICOL is 50.6 μM.

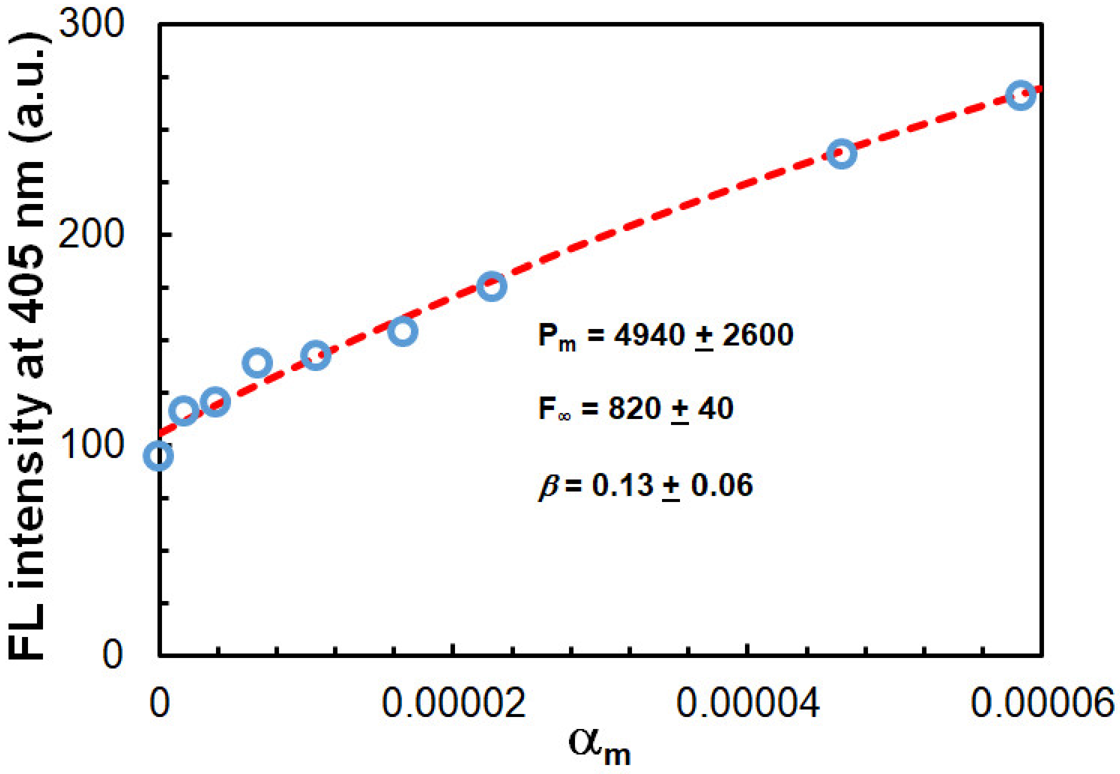

Figure S19. Variation of the fluorescence intensity (FL) at 405 nm with α_m. for the ICOL-Tween 80 micellar system.

6. Derivation of the determination of the association constant between ICOL and BSA

The association between the fluorophore (FL) and the BSA can be described by the equilibrium S33:

$$\text{FL} + \text{BSA} \overset{K}{\rightleftharpoons} \text{FL:BSA} \qquad \text{(S35)}$$

where K stands for the equilibrium constant.

A K can be expressed by eq. S34, where C_{FL}, C_{BSA} are the initial concentration of the fluorophore ICOL and BSA and $C_{FL:BSA}$ being the concentration of the FL-BSA associate.

$$K = \frac{C_{FL:BSA}}{(C_{FL} - C_{FL:BSA})(C_{BSA} - C_{FL:BSA})} \qquad \text{(S36)}$$

From eq. S36 the concentration of the FL-BSA associate ($C_{FL:BSA}$) can be given by eq. S37 as:

$$C_{FL:BSA} = \frac{K(C_{FL} + C_{BSA}) + 1 - \sqrt{[K(C_{FL} + C_{BSA}) + 1]^2 - 4K^2 C_{FL} C_{BSA}}}{2K} \quad \text{(S37)}$$

and the fluorescence intensity can now be expressed by eq. S38 (providing that the molar emissivity of the FL:BSA associate($\varepsilon_{FL:BSA}$) is much larger than that of the free fluorophore ICOL (ε_{FL}) at the given wavelength, i.e., $\varepsilon_{FL:BSA} >> \varepsilon_{FL}$

$$\text{Intensity} = \varepsilon_{FL:BSA} \left\{ \frac{K(C_{FL} + C_{BSA}) + 1 - \sqrt{[K(C_{FL} + C_{BSA}) + 1]^2 - 4K^2 C_{FL} C_{BSA}}}{2K} \right\} \text{(S38)}$$

Fitting eq. S38 to the experimental data, the value of K can be determined.

7. Quenching of ICOL and ICON solutions by pyridine (and Fig. S20-Fig. S22)

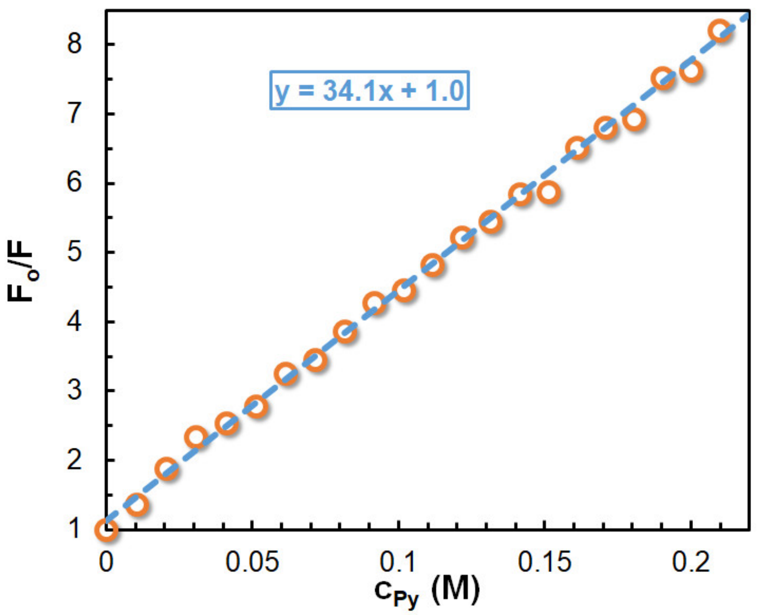

Figure S20. Stern-Volmer plot recorded at emission wavelength of 385 nm for the acetonitrile solution of ICON quenched by pyridine. The blue dashed lines are the fitted curve by eq. 5.

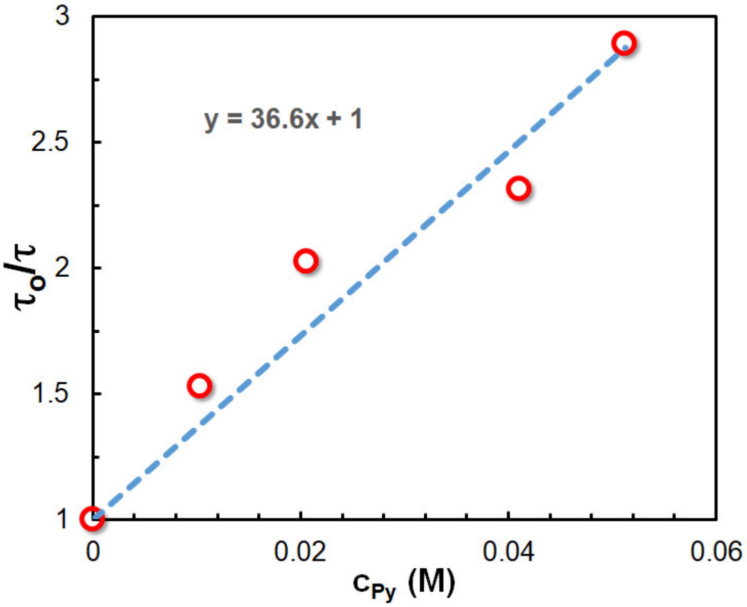

Figure S21. Stern-Volmer plot (τ_o/τ) recorded at emission wavelength of 385 nm for the acetonitrile solution of ICOL quenched by pyridine, where τ_o and τ is the fluorescence decay rate in the absence and presence of quencher, respectively. The concentration of ICOL was 1.7 μM and the excitation wavelength was 355 nm. The blue dashed lines are the fitted curve by eq. 5.

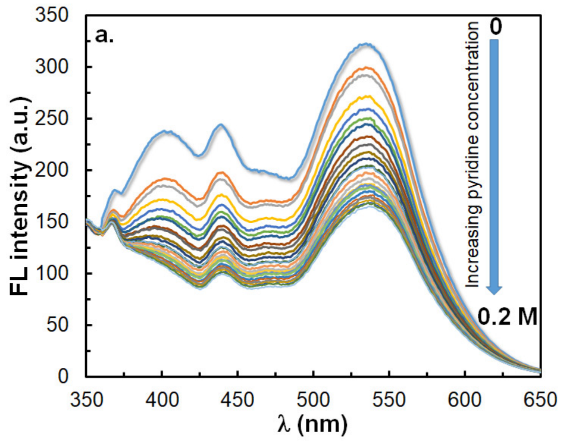

Figure S22. Variations of the emission spectra with the pyridine concentration (Py) for and aqueous solution of ICOL in the presence of Tween 80 at a concentration of 50 μM. (a). The concentration of ICOL is 5.9×10^{-5} M and the excitation wavelength is 327 nm. Stern-Volmer plots for Fig. S18a at 505 nm. The blue dashed lines are the fitted curves by eq. 6.
